# Functional Analysis and Genome Mining Reveal High Potential of Biocontrol and Plant Growth Promotion in Nodule-Inhabiting Bacteria Within *Paenibacillus polymyxa* Complex

**DOI:** 10.3389/fmicb.2020.618601

**Published:** 2021-01-18

**Authors:** Md. Arshad Ali, Yang Lou, Rahila Hafeez, Xuqing Li, Afsana Hossain, Ting Xie, Li Lin, Bin Li, Yanni Yin, Jianli Yan, Qianli An

**Affiliations:** ^1^State Key Laboratory of Rice Biology, Ministry of Agriculture Key Lab of Molecular Biology of Crop Pathogens and Insects, Zhejiang Province Key Laboratory of Biology of Crop Pathogens and Insects, Institute of Biotechnology, College of Agricultural and Biotechnology, Zhejiang University, Hangzhou, China; ^2^Hangzhou Academy of Agricultural Sciences, Hangzhou, China; ^3^Department of Plant Pathology, Bangabandhu Sheikh Mujibur Rahman Agricultural University, Gazipur, Bangladesh; ^4^Sugarcane Research Institute, Guangxi Academy of Agricultural Sciences, Nanning, China

**Keywords:** endophyte, *Fusarium*, fusaricidin, nitrogen fixation, plant growth promoting rhizobacteria

## Abstract

Bacteria belonging to the genus *Paenibacillus* were frequently isolated from legume nodules. The nodule-inhabiting *Paenibacillus* as a resource of biocontrol and plant growth-promoting endophytes has rarely been explored. This study explored the nodule-inhabiting *Paenibacillus*’ antifungal activities and biocontrol potentials against broad-spectrum important phytopathogenic fungi. We collected strains which were isolated from nodules of *Robinia pseudoacacia*, *Dendrolobium triangulare*, *Ormosia semicastrata*, *Cicer arietinum*, *Acacia crassicarpa*, or *Acacia implexa* and belong to *P. peoriae*, *P. kribbensis*, *P. endophyticus*, *P. enshidis*, *P. puldeungensis*, *P. taichungensis*, or closely related to *P. kribbensis*, or *P. anseongense*. These nodule-inhabiting *Paenibacillus* showed diverse antagonistic activities against five phytopathogenic fungi (*Fusarium graminearum*, *Magnaporthe oryzae*, *Rhizoctonia solani*, *Sclerotinia sclerotiorum*, and *Botrytis cinerea*). Six strains within the *P. polymyxa* complex showed broad-spectrum and potent activities against all the five pathogens, and produced multiple hydrolytic enzymes, siderophores, and lipopeptide fusaricidins. Fusaricidins are likely the key antimicrobials responsible for the broad-spectrum antifungal activities. The nodule-inhabiting strains within the *P. polymyxa* complex were able to epiphytically and endophytically colonize the non-host wheat plants, produce indole acetic acids (IAA), and dissolve calcium phosphate and calcium phytate. *P. peoriae* strains RP20, RP51, and RP62 could fix N_2_. *P. peoriae* RP51 and *Paenibacillus* sp. RP31, which showed potent plant colonization and plant growth-promotion competence, effectively control fungal infection *in planta*. Genome mining revealed that all strains (*n* = 76) within the *P. polymyxa* complex contain *ipdC* gene encoding indole-3-pyruvate decarboxylase for biosynthesis of IAA, 96% (*n* = 73) contain the *fus* cluster for biosynthesis of fusaricidins, and 43% (*n* = 33) contain the *nif* cluster for nitrogen fixation. Together, our study highlights that endophytic strains within the *P. polymyxa* complex have a high probability to be effective biocontrol agents and biofertilizers and we propose an effective approach to screen strains within the *P. polymyxa* complex.

## Introduction

The intense use of agrochemicals including chemical pesticides, synthetic fertilizers, and plant growth regulators to increase crop yields to meet the increasing food demand for increasing population has intensified the side effects of agrochemicals on agriculture, human health, and ecosystems (Lamichhane et al., 2016; Carvalho, 2017). Some microbes naturally in association with plants are able to produce antimicrobials and plant growth regulators, provide nutrients to plants by nitrogen fixation and phosphate solubilization, or induce plant systemic resistance to biotic and abiotic stresses, and thus can protect crops and promote crop growth (Grady et al., 2016; Alori et al., 2017; Cassán et al., 2020). The microbe-based biological control agents and biofertilizers are ecofriendly alternatives to control plant diseases and promote crop growth and are growingly developed to reduce the use of agrochemicals and support the sustainable agriculture (Grady et al., 2016; Alori et al., 2017; Cassán et al., 2020).

Endophytes are microbes that reside inside plants for part or full of their life cycle and are apparently not harmful to the host plants (Wilson, 1995; Hallman et al., 1997). Endophytes are apparently competent plant colonizers and adaptive to niches inside plants and may get sufficient nutrition and protection from plants; they may adapt to plant immune response and form close association or mutualistic relationship with plants and thus can be potent biocontrol agents and plant growth promoters (Rosenblueth and Martínez-Romero, 2006; Hardoim et al., 2015; Rybakova et al., 2016).

Endospore-forming bacteria belonging to the genus *Paenibacillus* are ubiquitous (Grady et al., 2016; Rybakova et al., 2016). Many *Paenibacillus* species are known for their production of antibiotics, especially polymyxins against Gram-negative bacteria and fusaricidins against fungi, oomycetes, and Gram-positive bacteria, and their nitrogen fixation (Xie et al., 2014; Grady et al., 2016; Rybakova et al., 2016). Like endospore-forming *Bacillus*, easy mass production in liquid culture, easy formulation, and long shelf-life support *Paenibacillus* to be promising biocontrol agents and biofertilizers (McSpadden Gardener, 2004; Grady et al., 2016; Rybakova et al., 2016).

*Paenibacillus* prefers to live in plant-associated habitats (McSpadden Gardener, 2004; Wang et al., 2020; Zhou et al., 2020) and are isolated frequently from legume root nodules (De Meyer et al., 2015; Pandya et al., 2015). Notably, some novel *Paenibacillus* species were identified based on strains isolated from legume nodules, such as *P. endophyticu*s from nodules of *Cicer arietinum* (Carro et al., 2013), *P. lupini* from nodules of *Lupinus albus* (Carro et al., 2014), *P. medicaginis* from nodules of alfalfa (Lai et al., 2015), *P. periandrae* from nodule of *Periandra mediterranea* (Menéndez et al., 2016), *P. prosopidis* from nodules of *Prosopis farcta* (Valverde et al., 2010), and “*P. enshidis*”(not validly published) from nodules of *Robinia pseudoacacia* (Yin et al., 2015). Recently, a *P. glycanilyticus* strain LJ121 isolated from yellow lupine (Ferchichi et al., 2019) was found to increase the total lipid content and modulate content of individual phenolic compounds in white lupine grains (Ferchichi et al., 2020). However, the roles of the nodule-inhabiting *Paenibacillus* to plants and plant-associated microbes are largely unknown.

Nodule-inhabiting *Paenibacillus* as a resource of biocontrol and plant growth-promoting endophytes has rarely been explored. The aim of this study was to screen nodule-inhabiting *Paenibacillus* strains having antagonistic activities against broad-spectrum phytopathogenic fungi and explore their antifungal mechanisms, plant colonization and plant growth-promotion competences, and biocontrol potentials. Five important fungal pathogens *Fusarium graminearum*, *Magnaporthe oryzae*, *Botrytis cinerea*, *Rhizoctonia solani*, and *Sclerotinia sclerotiorum* were selected as target pathogens because they have broad plant hosts, cause devastating damage to major staple food crops and economic crops, currently require high doses of chemical fungicides for control, and thus demand effective biocontrol agents to reduce the use of chemical fungicides.

## Materials and Methods

### *Paenibacillus* Strains and Fungal Pathogens

Twelve *Paenibacillus* strains (Table 1) were screened against phytopathogenic fungi. Five *Paenibacillus* strains RP20, RP31, RP43, RP51, and RP60 were isolated from surface sterilized root nodules of black locust (*Robinia pseudoacacia*) grown in Wuhan, Hubei Province, China. Nodules were surface sterilized in 70% (v/v) ethanol for 1 min and in 2% (w/v) sodium hypochlorite for 10 min and were rinsed with sterile water for six times. Surface-sterilized nodules were squashed and juices were streaked on yeast extract mannitol agar (YMA; per liter contains mannitol 10.0 g, yeast extract 0.8 g, K_2_HPO_4_ 0.25 g, KH_2_PO_4_ 0.25 g, MgSO_4_⋅7H_2_O 0.2 g, NaCl 0.1 g, and agar 15.0 g; pH 7.0). After incubation at 30°C for 3 days, single colonies were purified on YMA. Four *Paenibacillus* strains CFCC 1854, CFCC 1865, CFCC 13938, and CFCC 1991 isolated from nodules were obtained from China Forestry Culture Collection Center. *P. endophyticus* type strain CCTCC AB 2014195^*T*^ (=PECAE04^*T*^ = LMG 27297^*T*^) (Carro et al., 2013) and “*P. enshidis*” type strain CCTCC AB 2013275^*T*^ (=RP-207^*T*^ = KCTC 33519^*T*^) (Yin et al., 2015) were obtained from China Center for Type Culture Collection. *P. peoriae* type strain CGMCC 1.3761^*T*^ isolated from soil was obtained from China General Microbiological Culture Collection Center. The *Paenibacillus* strains were stored in 15% (v/v) glycerol at −80°C and recovered on LB agar (per liter contains yeast extract 5 g, tryptone 10 g, NaCl 10 g, and agar 15 g; pH 7.0) at 30°C.

**TABLE 1:** *Paenibacillus* strains used in this study.

**Organism**	**Strain**	**Isolation source**	**Plant host**	**16S rRNA gene accession no.**
*Paenibacillus peoriae*	RP20	Nodule	*Robinia pseudoacacia*	MN715870
*Paenibacillus peoriae*	RP51	Nodule	*Robinia pseudoacacia*	MN715872
*Paenibacillus peoriae*	RP62	Nodule	*Robinia pseudoacacia*	MN715875
*Paenibacillus peoriae*	CFCC 1854	Nodule	*Dendrolobium triangulare*	MT093458
*Paenibacillus* sp.	RP31	Nodule	*Robinia pseudoacacia*	MN715871
*Paenibacillus kribbensis*	CFCC 1865	Nodule	*Ormosia semicastrata*	MT093459
*Paenibacillus endophyticus*	CCTCC AB 2014195^*T*^	Nodule	*Cicer arietinum*	KC447384
*Paenibacillus enshidis*	CCTCC AB 2013275^*T*^	Nodule	*Robinia pseudoacacia*	KF862945
*Paenibacillus puldeungensis*	CFCC 13938	Nodule	*Acacia crassicarpa*	MT093460
*Paenibacillus taichungensis*	CFCC 1991	Nodule	*Acacia implexa*	MT093461
*Paenibacillus* sp.	RP43	Nodule	*Robinia pseudoacacia*	MT040706
*Paenibacillus peoriae*	CGMCC 1.3761^*T*^	Soil	Not applicable	AJ320494

Mycelia of five phytopathogenic fungi *F. graminearum* strain PH-1 (Yin et al., 2018) *M. oryzae* strains Guy11 (Que et al., 2020), *B. cinerea* strain B05.10 (Shao et al., 2016), *R. solani* strain TTZF-1 (Liu et al., 2012), and *S. sclerotiorum* strain 7-3 (Xu et al., 2012) were stored in 30% (v/v) glycerol at −80°C at the Institute of Biotechnology, Zhejiang University and recovered on potato dextrose agar (PDA) (per liter contains potato infusion 200 g, glucose 20 g, and agar 15 g) at 28°C.

### Amplification and Phylogenetic Analysis of 16S rRNA Gene Sequences of *Paenibacillus* Strains

Bacterial 16S rRNA gene sequences were amplified with the primers 27F (5′-AGAGTTTGATCCTGGCTCAG-3′) and 1492R (5′-GGTTACCTTGTTACGACTT-3′) from colonies grown on the LB agar as previously described (Ali et al., 2020). Non-type *Paenibacillus* strains were identified based on the identity between their16S rRNA gene sequences and those of the type strains at the EzBioCloud^1^ and the phylogenetic status of their 16S rRNA gene sequences. Nucleotide sequences were aligned using the MUSCLE program; positions containing gaps and missing data were eliminated; final 1319 positions were constructed to a phylogenetic tree with the Neighbor-Joining method and the Kimura 2-parameter model integrated in the MEGA5 software (Tamura et al., 2011).

### Screening Antagonistic *Paenibacillus* Against Fungal Pathogens

*Paenibacillus* antagonistic activities against fungal pathogens were examined by the bacteria-fungi confrontation assay on PDA. A 5-mm mycelial plug from the edge of a fresh 7-d fungal colony on PDA was transferred to the center of a fresh PDA in a 90-mm Petri dish. *Paenibacillus* strains were cultured in the LB broth at 30°C and 200 rpm for 48 h. Bacterial suspension was adjusted to 1 × 10^8^ CFU ml^–1^ and 5 μl of the bacterial suspension was inoculated at 25 mm away from the fungal plug on the PDA plate. A fungal mycelial plug alone on the PDA plate was used as control. The PDA plates were kept at 28°C for 7 days. *Paenibacillus* antagonistic activities were measured by inhibition of mycelial growth according to Riungu et al. (2008). Four replications were tested for each *Paenibacillus* strain and the experiment was repeated three times.

### Assay of Hydrolytic Enzyme Activities From *Paenibacillus* Against Broad-Spectrum Fungi

Hydrolytic enzyme activities of the *Paenibacillus* strains inhibiting all the five tested fungal pathogens were assessed using minimal salt agar media supplemented with the enzyme substrate. The minimal salt medium per liter contains (NH_4_)_2_SO_4_ 1 g, KH_2_PO_4_ 0.2 g, K_2_HPO_4_ 1.6 g, MgSO_4_⋅7H_2_O 0.2 g, NaCl 0.1 g, FeSO_4_⋅7H_2_O 0.01 g, and CaCl_2_⋅2H_2_O 0.02 g. The medium for assessing chitinase activity was supplemented with 1% (w/v) colloidal chitin and 1.5% (w/v) agar and adjusted to the final pH 7.0 and sterilized by autoclaving at 121°C for 15 min. The medium for assessing β*-*1,3*-*glucanase activity was supplemented with 0.5 g of glucose, 6.7 g of yeast extract, 60 mg of aniline blue and 4 g of pachyman powder (Megazyme Ltd., Wicklow, Ireland) per liter. The pH was adjusted to 6.8 and 1.2 g of agar was added before autoclaving at 121°C for 10 min (Mahasneh and Stewart, 1980). The medium for assessing protease was supplemented with 1% (w/v) 1% (w/v) casein. Bacterial suspension was adjusted to 1 × 10^8^ CFU ml^–1^ and 5 μl of the bacterial suspension was inoculated onto the agar plates and incubated at 28°C for 2–3 days. The chitin plates were stained with 0.1% (w/v) Congo red and washed with 1% NaCl. Bacterial hydrolytic enzyme activities were indicated by hydrolysis zones around the bacterial colonies.

Colloidal chitin was prepared according to Souza et al. (2009). Five grams of chitin powder (J&K Scientific Ltd., Beijing, China) was added slowly to 60 ml of concentrated hydrochloric acid in an Erlenmeyer flask and kept with shaking at 180 rpm at 37°C for 1 h. The mixture was filtered through glass wool and the filtrate was added to a 200 ml of 50% (v/v) ethanol with vigorous stirring. The precipitate was transfer to a glass funnel with filter paper and washed with sterile distilled water until the colloidal chitin reached a neutral pH 7. The colloidal chitin retained on the filter paper was collected, weighed and stored in dark at 4°C before use.

### Assay of Siderophore Production From *Paenibacillus* Against Broad-Spectrum Fungi

Siderophore production from the *Paenibacillus* strains inhibiting all the five tested fungal pathogens was assessed using the blue medium containing chrome azurol S (Schwyn and Neilands, 1987). Chelation of iron by siderophores was indicated by color changes from blue to purple (catechol type) or orange (hydroxamate type) in and around the bacterial colonies.

### MALDI-TOF-MS Analysis for Lipopeptides From *Paenibacillus* Against Broad-Spectrum Fungi

Lipopeptides were detected by matrix-assisted laser desorption ionization-time of flight-mass spectrometry (MALDI-TOF-MS) (Masum et al., 2018). Bacteria were grown on LB agar at 30°C for 48 h. A bacterial colony was suspended in a matrix solution [α-cyano-4-hydroxycinnamic acid (10 mg ml^–1^) in 30% (v/v) of acetonitrile and 70% (v/v) of 0.1% (w/v) TFA in water]. The suspension (1 μl) was spotted onto a target plate, air dried, and detected with a reflection-positive mode in the mass spectral range from 300 to 3000 Da using an Ultraflextreme MALDI-TOF mass spectrometer (Bruker, Bremen, Germany) equipped with a 355 nm nitrogen laser.

### Scanning Electron Microscopy (SEM) and Transmission Electron Microscopy (TEM)

Blocks of 7-day-old fungal mycelia were taken from the edge of *Fusarium graminearum* mycelia grown on PDA alone or confrontation with *P. peoriae* RP51 and prepared for electron microscopy. The blocks were immersed in 2.5% (v/v) glutaraldehyde in 0.1 M phosphate buffer (pH 7.0) for 4 h, washed with the phosphate buffer for 15 min three times, then immersed in 1% (w/v) OsO_4_ in the phosphate buffer for 2 h and washed three times with the phosphate buffer. The blocks were dehydrated in a graded series of ethanol for 15 min at each step and then in absolute acetone for 20 min twice. For SEM, the blocks were dehydrated in an HCP-2 critical point dryer (Hitachi, Tokyo, Japan) and then coated with gold-palladium in an E-1010 ion sputter (Hitachi, Tokyo, Japan) and observed with an Gemini SEM 300 scanning electron microscope (Carl Zeiss, Jena, Germany). For TEM, the dehydrated blocks were infiltrated with graded series of Spurr’s resin mixed with absolute acetone and then the Spurr’s resin overnight. The blocks embedded in the Spurr’s resin were polymerized at 70°C for 12 h. The specimen was sectioned with an EM UC7 ultratome (Leica Microsystems, Vienna, Austria). The ultrathin sections were stained with uranyl acetate and lead citrate and then observed with an H-7650 transmission electron microscope (Hitachi, Tokyo, Japan).

### Assay of Plant Growth Promotion and Plant Colonization by *Paenibacillus* Against Broad-Spectrum Fungi

Wheat seeds (cv. Jimai22) were surface-sterilized by 70% ethanol for 1 min and 3% sodium hypochlorite for 6 min, and washed with sterile water for six times. Seeds were imbibed in sterile water overnight. The *Paenibacillus* strains against broad-spectrum fungi were grown in LB broth at 30°C and 200 rpm for 48 h and then washed with sterile water and suspended to about 1 × 10^8^ CFU ml^–1^. The seeds (*n* = 100) were immersed in 10 ml of each bacterial suspension for 4 h or in sterile water as uninoculated control. The seeds were dried in a laminar flow hood and 15 seeds were placed on two layers of sterile 125-mm Whatman filter paper moistened with 10 ml of sterile water in a 150-mm Petri dish for germination and growth at 25°C under 16-h light and 8-h dark photoperiod. At 9 days after inoculation, root length, shoot length, fresh weight, and dry weight of the seedlings were measured. To determine epiphytic root colonization of the *Paenibacillus* strains, bacteria adhere to roots were washed off in 3 ml of sterile water and serially diluted; 100 μl of the bacterial suspensions were spread on LB agar. To determine endophytic colonization of the *Paenibacillus* strains in roots and stems, roots and stems were surface-sterilized by 70% ethanol for 1 min and 1% sodium hypochlorite for 2 min, washed with sterile water for six times. The sterilization efficacy was determined by no bacterial growth from the last washed water. The surface-sterilized roots and stems were ground in sterile water. The homogenate suspensions were serially diluted; 100 μl of the diluted suspensions were spread on LB agar. The LB agar plates were kept at 30°C for 3 days and bacterial colonies showing the colony morphology of the inoculated strain were counted. The experiments were done with three replicates and repeated three times.

### *In planta* Assay of Disease Control by *Paenibacillus* Against *Fusarium*

Fusarium root rot (FRR) and Fusarium foot rot (FFR) on wheat seedlings were used as the pathosystem (Colombo et al., 2019) to determine the biocontrol potential by selected *Paenibacillus* against broad-spectrum fungi *in planta*. Wheat seeds were surface-sterilized and inoculated by *Paenibacillus*, and prepared for germination as described above. Control seeds were treated with sterile water. Ten seeds were placed on the filter papers in a 150-mm Petri dish. When the primary roots of the seedlings reached about 30 mm, a 5-mm mycelial plug taken from the edge of an actively growing *F. graminearum* was put upside down on a primary root at a 10 mm distance from the seed. A sterile PDA plug was used as negative control. At 4 days after fungal inoculation, the extension (length) of the necrosis on the root (FRR) was measured (Supplementary Figure 1A). Inhibition of FRR by *Paenibacillus* was determined using the formula (Control necrosis – *Paenibacillus*-treated necrosis)/Control necrosis × 100 (Colombo et al., 2019). At 6 days after fungal inoculation, FFR was measured by scoring the symptoms at the crown level (0 = symptomless; 1 = slightly necrotic; 2 = moderately necrotic; 3 = severely necrotic; 4 = completely necrotic) (Supplementary Figure 1B). The FFR disease severity was determined using the formula [Σ (Disease grade × number of plants in each grade)/(Total number of plants × highest disease grade)] × 100. Inhibition of FFR by *Paenibacillus* was determined using the formula (Control disease severity – *Paenibacillus*-treated disease severity)/Control disease severity × 100 (Colombo et al., 2019).

### Assays of Plant Growth-Promotion Traits of *Paenibacillus* Against Broad-Spectrum Fungi

Indole acetic acid (IAA) production by *Paenibacillus* was determined using the colorimetric assay developed by Sarwar and Kremer (1995). *Paenibacillus* strains were grown in LB broth supplemented with L-tryptophan (100 μg ml^–1^) (BBI Life Science, Shanghai, China) in dark at 30°C for 3 days. After centrifugation, 150 μl of the culture supernatant was added into wells of 96-well microplate followed by addition of 100 μ1 of the Salkowski reagent and incubation in dark for 30 min. IAA (1 mg ml^–1^) (BBI Life Science, Shanghai, China) was diluted to 10 μg ml^–1^ to 50 μg ml^–1^ as standards and added into the wells. Each IAA standard and bacterial culture supernatant was tested in three replicate wells. After the 30-min reaction, the absorbance of the pink product was measured at 530 nm using a SpectraMax^®^ Plus 384 Microplate Spectrophotometer (Molecular Devices Corporation, Sunnyvale, CA, United States). An IAA standard curve was generated and the IAA in the bacterial culture supernatant was quantified accordingly (Sarwar and Kremer, 1995).

Bacterial solubilization of phosphate was determined by the assay on the NBRIP media (per liter contains glucose 10 g, MgCl_2_.6H_2_O 5 g, MgSO_4_.7H_2_O 0.25 g, KCl 0.2 g, (NH_4_)_2_SO_4_ 0.1 g, agar 15 g; pH 7.0) containing 0.5% (w/v) calcium phosphate, ferric phosphate, or calcium phytate according to Nautiyal (1999) and Bashan et al. (2013).

Bacterial nitrogen fixation was determined by growth on nitrogen-free Jensen’s agar medium (per liter contains sucrose 20 g, K_2_HPO_4_ 1 g, MgSO_4_.7H_2_O 0.5 g, NaCl 0.5 g, FeSO_4_ 0.1 g, Na_2_MoO_4_ 5 mg, CaCO_3_ 2 g) and PCR amplification of partial *nifH* gene. Colony PCR was done with the forward primer (5′-GGCTGCGATCCVAAGGCCGAYTCVACCCG-3′) and the reverse primer (5′-CTGVGCCTTGTTYTC GCGGATSGGCATGGC-3′) designed by Ding et al. (2005) and a 2 × PCR Master Mix (TSINGKE Biological Technology, Beijing, China). The PCR program was pre-denaturation at 98°C for 5 min, 30 cycles of denaturation at 98°C for 10 s, annealing at 68°C for 10 s, and elongation at 72°C for 10 s, and a final elongation at 72°C for 5 min. PCR products (323 bp) were detected by agarose gel electrophoresis with 1% (w/v) agarose.

### Genome Mining

Gene clusters for biosynthesis of secondary metabolites (fusaricidins and siderophores) were mined from target whole genome sequences deposited in the NCBI database^2^ as at 2020-07-30 using the antiSMASH 5.0 pipeline (Blin et al., 2019). Protein names “nitrogenase,” “*ilvB*,” and “1-aminocyclopropane-1-carboxylate deaminase” (ACC deaminase) were searched from the annotated protein coding genes (CDS) of the target whole genome sequence (Protein Table for the target organism) in the NCBI database (see text footnote 2). Gene *ilvB* encoding the biosynthetic-type acetolactate synthase large subunit is the gene *ipdC* encoding indole-3-pyruvate decarboxylase for the biosynthesis of indole acetic acid (IAA) via the IPyA pathway (Xie et al., 2016).

### Statistical Analysis

All experiments were performed in completely randomized design. All the values were presented as mean ± standard error of at least three replications. One-way analysis of variance (ANOVA) was used to analyze the data following *post hoc* multiple comparisons using SPSS 16.0 software (SPSS Inc., Chicago, IL, United States). Least significant difference test was done to separate the treatments.

## Results

### Nodule-Inhabiting Strains Within *Paenibacillus polymyxa* Complex Showed Broad-Spectrum Antifungal Activities

**FIGURE 1:**
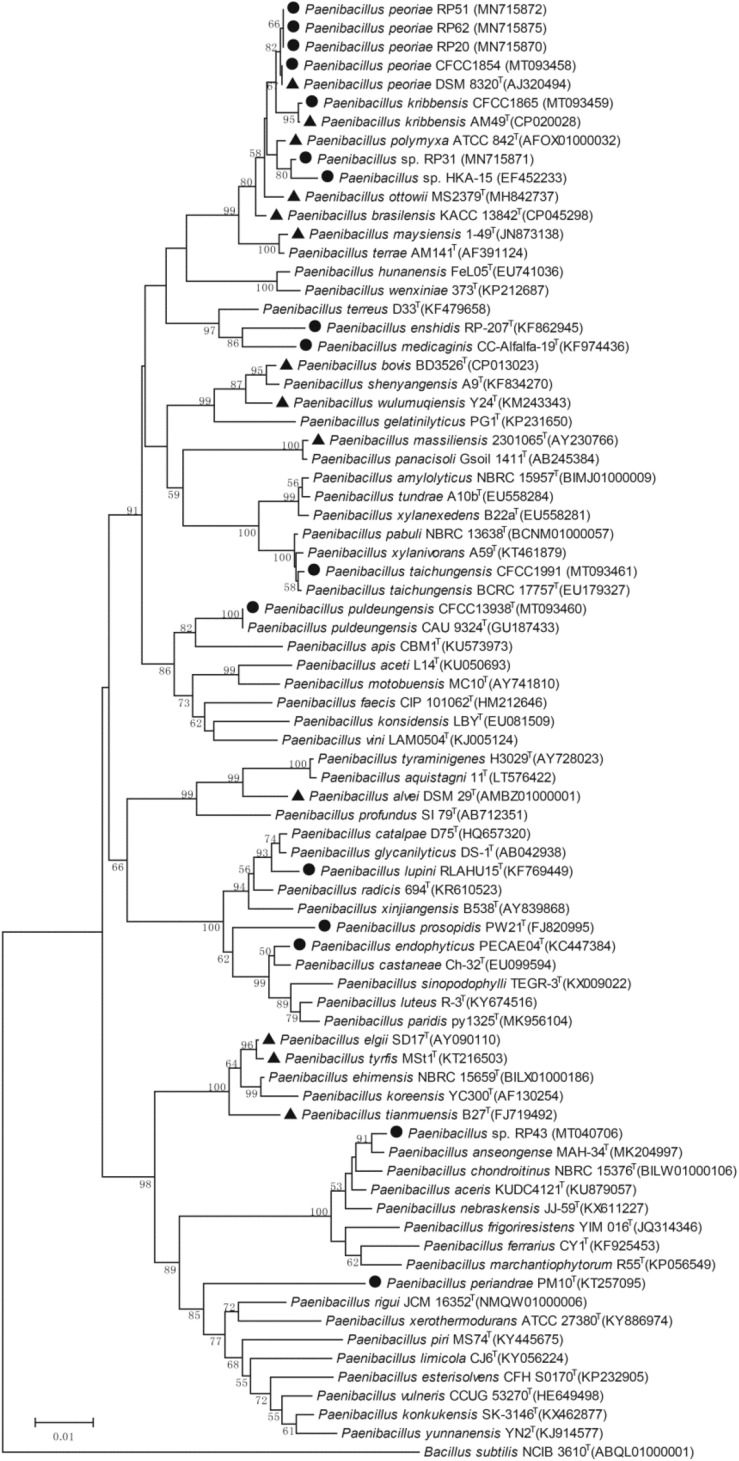
Neighbor-joining phylogenetic tree based on 16S rRNA gene sequences (1319 positions) of nodule-inhabiting *Paenibacillus* strains (•), *Paenibacillus* strains containing *fus* gene cluster (▲), and their relatives. *Bacillus subtilis* NCIB 3610^*T*^ was used as outgroup. The percentages of replicate trees (>50%) in which the associated taxa clustered together in the bootstrap test (1000 replicates) are shown at the nodes. The GenBank accession numbers of the 16S rRNA gene sequences are indicated in brackets. The scale bar indicates 0.01 substitutions per site.

The phylogenetic tree of the 16S rRNA gene sequences of the nodule-inhabiting *Paenibacillus* strains and their relatives showed that *P. polymyxa* (the type species of the genus *Paenibacillus*), *P. peoriae*, *P. kribbensis*, *P. ottowii*, *P. brasilensis*, *P. terrae*, and “*P. maysiensis*” formed a monophyletic complex (*Paenibacillus polymyxa* complex) (Figure 1). Strains RP20, RP51, and RP62 isolated from nodules of *Robinia pseudoacacia* and strain CFCC 1854 isolated from *Dendrolobium triangulare* were classified to *P. peoriae* because their 16S rRNA gene sequences and that of *P. peoriae* type strain have identities above 99.6% and phylogenetically grouped together (Figure 1). Strain CFCC 1865 isolated from nodules of *Ormosia semicastrata* was classified to *P. kribbensis* because its 16S rRNA gene sequence and that of *P. kribbensis* type strain AM49^*T*^ have an identity of 99.7% and phylogenetically grouped together. Strain RP31 isolated from nodules of *Robinia pseudo acacia* and strain HKA-15 isolated from nodules of soybean (Annapurna et al., 2013) may belong to a same species because their 16S rRNA gene sequences showed identities of 99.2% and phylogenetically grouped together; they may belong to *P. kribbensis* or a novel species closely related to *P. kribbensis* because their 16S rRNA gene sequences showed identities about 98.8% to that of *P. kribbensis* AM49^*T*^ and phylogenetically grouped close to *P. kribbensis* AM49^*T*^ (Figure 1).

“*Paenibacillus enshidis*” CCTCC AB 2013275^*T*^ (=RP-207^*T*^) isolated from *Robinia pseudoacacia* (Yin et al., 2015) and *P. medicaginis* CC-Alfalfa-19^*T*^ isolated from nodules of alfalfa (Lai et al., 2015) are phylogenetic neighbors within a cluster closely related to the cluster containing the *P. polymyxa* complex (Figure 1). Strain CFCC 1991 isolated from nodules of *Acacia implexa* was classified to *P. taichungensis* because its 16S rRNA gene sequence and that of *P. taichungensis* type strain have an identity of 99.8% and phylogenetically grouped together within a standalone phylogenetic cluster (Figure 1). Strain CFCC 13938 isolated from nodules of *Acacia crassicarpa* was classified to *P. puldeungensis* because its partial 16S rRNA gene sequence (1395 bp) is identical to that of *P. puldeungensis* type strain CAU 9324^*T*^ within a standalone phylogenetic cluster (Figure 1).

*Paenibacillus endophyticus* PECAE04^*T*^ (=CCTCC AB 2014195^*T*^) isolated from nodules of *Cicer arietinum* (Carro et al., 2013), *P. lupini* RLAHU15^*T*^ isolated from nodules of *Lupinus albus* (Carro et al., 2014), and *P. prosopidis* PW21^*T*^ isolated from nodules of *Prosopis farcta* (Valverde et al., 2010) were within a standalone phylogenetic cluster (Figure 1).

Strain RP43 isolated from nodules of *Robinia pseudoacacia* may belong to *P. anseongense* or a novel species closely related to *P. anseongense* because its 16S rRNA gene sequence and that of *P. anseongense* type strain have an identity of 99.0% and phylogenetically grouped together (Figure 1). Strain RP43 is relatively close to *P. periandrae* PM10^*T*^ isolated from nodules of *P. mediterranea* (Menéndez et al., 2016).

The nodule-inhabiting strains within the *P. polymyxa* complex inhibited the growth of all the five tested fungal pathogens. *P. peoriae* strains RP20, RP51, and RP62, *P. kribbensis* CFCC 1865, and *Paenibacillus* sp. RP31 showed above 60% inhibition on the growth of the five fungal pathogens. *P. peoriae* CFCC 1854 showed relatively lower inhibition (below 60%) on *F. graminearum, B. cinerea*, *R. solani*, and *S. sclerotiorum*. RP20, RP51, RP62, RP31, and CFCC 1865 showed similar inhibition on *M. oryzae*. Strain RP51 showed the highest inhibition on *F. graminearum*, *B. cinerea*, and *S. sclerotiorum*. Strain RP31 showed the highest inhibition on *B. cinerea* and *R. solani*. In contrast, *P. peoriae* type strain CGMCC 1.3761^*T*^, which was isolated from soil, inhibited only the growth of *M. oryzae* at a relatively lower extent than that by the six nodule-inhabiting strains within the *P. polymyxa* complex (Table 2 and Supplementary Figure 2). *P. endophyticus* CCTCC AB 2014195^*T*^ inhibited the growth of *F. graminearum, M. oryzae*, and *R. solani* but not *B. cinerea* and *S. sclerotiorum*. “*P. enshidis*” CCTCC AB 2013275^*T*^ and *Paenibacillus* sp. RP43 inhibited only the growth of *F. graminearum*. *P. puldeungensis* CFCC 13938 and *P. taichungensis* CFCC 1991 did not inhibit fugal growth (Table 2).

**TABLE 2:** Inhibition of fungal growth by *Paenibacillus* strains.

**Organisms**	**Strains**	Inhibition of fungal growth (%)*				
		
		***Fusarium graminearum***	***Magnaporthe oryzae***	***Botrytis cinerea***	***Rhizoctonia solani***	***Sclerotinia sclerotiorum***
*Paenibacillus peoriae*	RP20	70.7 ± 0.0^b^	65.8 ± 1.4^ab^	65.9 ± 1.0^b^	64.2 ± 0.6^b^	64.0 ± 0.6^b^
*Paenibacillus peoriae*	RP51	75.0 ± 1.2^a^	68.2 ± 1.0^a^	67.7 ± 1.1^ab^	61.0 ± 2.6^c^	67.1 ± 1.6^a^
*Paenibacillus peoriae*	RP62	68.9 ± 0.6^bcd^	67.1 ± 1.6^a^	66.5 ± 1.2^b^	63.4 ± 0.7^b^	62.8 ± 0.6^b^
*Paenibacillus peoriae*	CFCC 1854	59.2 ± 1.2^e^	68.9 ± 2.1^a^	59.1 ± 0.6^c^	44.5 ± 1.8^d^	41.4 ± 1.0^d^
*Paenibacillus* sp.	RP31	70.1 ± 0.6^bc^	65.9 ± 0.0^ab^	69.5 ± 0.7^a^	68.9 ± 1.1^a^	62.8 ± 0.6^b^
*Paenibacillus kribbensis*	CFCC 1865	67.7 ± 0.6^cd^	68.9 ± 0.6^a^	65.9 ± 2.5^b^	64.7 ± 0.7^b^	61.0 ± 0.0^c^
*Paenibacillus endophyticus*	CCTCC AB 2014195^T^	61.0 ± 1.0^e^	57.9 ± 2.3^c^	0^f^	31.7 ± 1.0^e^	0^e^
*Paenibacillus enshidis*	CCTCC AB 2013275^T^	46.4 ± 1.4^f^	0^d^	0^f^	0^f^	0^e^
*Paenibacillus* sp.	RP43	67.1 ± 0.7^d^	0^d^	0^f^	0^f^	0^e^
*Paenibacillus puldeungensis*	CFCC 13938	0^g^	0^d^	0^f^	0^f^	0^e^
*Paenibacillus taichungensis*	CFCC 1991	0^g^	0^d^	0^f^	0^f^	0^e^
*Paenibacillus peoriae*	CGMCC 1.3761^T^	0^g^	54.3 ± 1.2^c^	0^f^	0^f^	0^e^

*Values are mean ± SE obtained from four replication of each treatment. Different letters following the values in the same column indicate significant differences between the treatments at *p* < 0.05.

### Nodule-Inhabiting Strains Within *Paenibacillus polymyxa* Complex Produced Hydrolytic Enzymes, Siderophores, and Fusaricidins

Six nodule-inhabiting *Paenibacillus* strains within the *P. polymyxa* complex, *P. peoriae* RP20, RP51, RP62, and CFCC 1854, *P. kribbensis* CFCC 1865, and *Paenibacillus* sp. RP31, showed antagonistic activities against all the five tested fungal pathogens and thus were noted as *Paenibacillus* against broad-spectrum fungi. To know the mechanisms of the broad-spectrum antifungal activities of these nodule-inhabiting strains within the *P. polymyxa* complex, some known antifungal substances (hydrolytic enzymes, siderophores, and fusaricidins) were examined. *P. peoriae* strains CGMCC 1.3761^*T*^ within the *P. polymyxa* complex was also examined for comparison.

**FIGURE 2:**
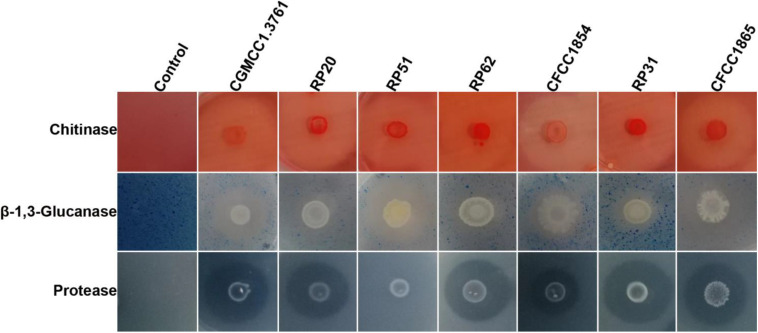
Agar plate assays show hydrolysis of chitin, β-1,3-glucan, and casein by chitinase, β-1,3-glucanase, and protease from *P. peoriae* CGMCC 1.3761^*T*^ and nodule-inhabiting strains within *Paenibacillus polymyxa* complex.

All the seven strains within the *P. polymyxa* complex showed activities of chitinase, β-1,3-glucanase, and protease except that *P. peoriae* RP51 showing the highest inhibition on *F. graminearum*, *B. cinerea*, and *S. sclerotiorum* did not show protease activity (Figure 2). They show similar chitinase activities. *P. peoriae* CGMCC 1.3761^*T*^ and RP51, and *P. kribbensis* CFCC 1865 showed relatively stronger β-1,3-glucanase activities (Figure 2). The weak antifungal strain *P. peoriae* CGMCC 1.3761^*T*^ showed similar chitinase activity and β-1,3-glucanase activity as the potent antifungal strain RP51. Seemingly, the hydrolytic enzyme activities detected from these strains were not consistent with the extent of their antifungal activities. Therefore, the hydrolytic enzymes may not contribute to the broad-spectrum antifungal activities of the nodule-inhabiting strains within the *P. polymyxa* complex.

**FIGURE 3:**
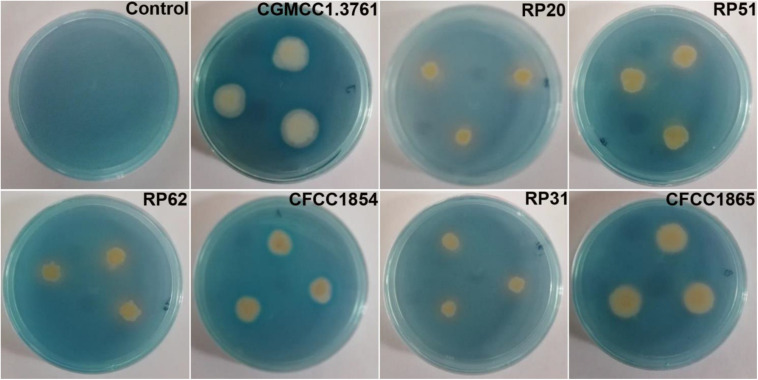
Chrome azurol S assay shows color changes from blue to light purple or orange indicating production of catechol type or hydroxamate type siderophores from *P. peoriae* CGMCC 1.3761^*T*^ and nodule-inhabiting strains within *Paenibacillus polymyxa* complex.

All the seven strains grown on the iron limited medium produced siderophores detected by the chrome azurol S assay (Figure 3). *P. peoriae* RP20, RP51, and RP62, and *Paenibacillus* sp. RP31 shown potent antifungal activities on PDA appeared to produce relatively more siderophores (Figure 3). However, the production of siderophores may not contribute to the broad-spectrum antifungal activities detected on the iron-sufficient PDA.

MALDI-TOF-MS detected fusaricidins in the range of *m/z* 860–1050 (Vater et al., 2015, 2017) from all these strains within the *P. polymyxa* complex (Table 3 and Supplementary Figure 3). *P. peoriae* RP20, RP51 and RP62 produced diverse fusaricidins including Fusaricidin A, Fusaricidin B, Fusaricidin C, Fusaricidin D, Fusaricidin A(C17)/E(C15), and LI-F08b. *P. peoriae* RP51 and RP62 also produced fusaricidin LI-F05b. *Paenibacillus* sp. RP31 produced Fusaricidin A, Fusaricidin B, Fusaricidin C, Fusaricidin D, and Fusaricidin A(C17)/E(C15). *P. kribbensis* CFCC 1865 produced Fusaricidin A, Fusaricidin B, Fusaricidin A(C_17)_/E(C_15_), LI-F08b and LI-F05b. *P. peoriae* CFCC1854, which showed relatively weaker antifungal activities against *Fusarium graminearum, Botrytis cinerea, Rhizoctonia solani* and *Sclerotinia sclerotiorum*, produced less diverse fusaricidins including LI-F03a and three unknown fusaricidin-like compounds. *P. peoriae* CGMCC 1.3761^*T*^, which inhibited only the growth of *Magnaporthe oryzae*, produced three unknown fusaricidin-like compounds (Table 3 and Supplementary Figure 3). In consistent with the production of fusaricidins, a nonribosomal peptide synthetase gene cluster most similar to the gene cluster for biosynthesis of fusaricidin B was detected using antiSMASH from the genome of the *P. peoriae* KCTC 3763^*T*^ (=CGMCC 1.3761^*T*^).

**TABLE 3:** Lipopeptides identified by MALDI-TOF MS from strains within the *Paenibacillus polymyxa* complex.

**Mass peaks (m/z)**	**Lipopeptides**	**References**
*P. peoriae* CGMCC 1.3761^*T*^		
861.2	Unknown fusaricidin	Vater et al. (2015)
877.2	Unknown fusaricidin	Vater et al. (2015)
893.2	Unknown fusaricidin	Vater et al. (2015)
994.2	Val-7 C_13_ surfactin [M+H]^+^	Vater et al. (2003)
*P. peoriae* RP20		
868.0	Fusaricidin A [M+H]^+^	Vater et al. (2015)
883.4	Fusaricidin A [M+H]^+^	Kajimura and Kaneda (1996); Vater et al. (2015)
897.4	Fusaricidin B [M+H]^+^	Kajimura and Kaneda (1997); Vater et al. (2015)
905.4	Fusaricidin A [M+Na]^+^	Vater et al. (2015)
919.4	Fusaricidin B [M+Na]^+^	Vater et al. (2015)
947.4	Fusaricidin C [M+H]^+^	Kajimura and Kaneda (1997); Vater et al. (2015)
935.4	Fusaricidin B [M+K]^+^	Vater et al. (2015)
961.4	Fusaricidin D [M+H]^+^	Kajimura and Kaneda (1997); Vater et al. (2015)
911.5	Fusaricidin A(C_17)_/E(C_15_) [M+H]^+^	Vater et al. (2017)
925.5	Fusaricidin LI-F08b [M+H]^+^	Vater et al. (2015)
*P. peoriae* RP51		
868.2	Fusaricidin A [M+H]^+^	Vater et al. (2015)
883.7	Fusaricidin A [M+H]^+^	Kajimura and Kaneda (1996); Vater et al. (2015)
897.7	Fusaricidin B [M+H]^+^	Kajimura and Kaneda (1997); Vater et al. (2015)
905.7	Fusaricidin A [M+Na]^+^	Vater et al. (2015)
919.7	Fusaricidin B [M+Na]^+^	Vater et al. (2015)
947.8	Fusaricidin C [M+H]^+^	Vater et al. (2015)
961.8	Fusaricidin D [M+H]^+^	Kajimura and Kaneda (1997); Vater et al. (2015)
911.8	Fusaricidin A(C_17)_/E(C_15_) [M+H]^+^	Vater et al. (2017)
925.8	Fusaricidin LI-F08b [M+H]^+^	Vater et al. (2015)
933.8	Fusaricidin LI-F05b [M+Na]^+^	Vater et al. (2015)
*P. peoriae* RP62		
868.0	Fusaricidin A [M+H]^+^	Vater et al. (2015)
883.7	Fusaricidin A [M+H]^+^	Kajimura and Kaneda (1996); Vater et al. (2015)
897.6	Fusaricidin B [M+H]^+^	Kajimura and Kaneda (1997); Vater et al. (2015)
905.6	Fusaricidin A [M+Na]^+^	Vater et al. (2015)
919.5	Fusaricidin B [M+Na]^+^	Vater et al. (2015)
947.6	Fusaricidin C [M+H]^+^	Kajimura and Kaneda (1997); Vater et al. (2015)
961.6	Fusaricidin D [M+H]^+^	Kajimura and Kaneda (1997); Vater et al. (2015)
911.6	Fusaricidin A(C_17)_/E(C_15_) [M+H]^+^	Vater et al. (2017)
925.6	Fusaricidin LI-F08b [M+H]^+^	Vater et al. (2015)
933.6	Fusaricidin LI-F05b [M+Na]^+^	Vater et al. (2015)
*P. peoriae* CFCC 1854		
940.6	Unknown fusaricidin	Vater et al. (2015)
954.6	Fusaricidin LI-F03a [M+H]^+^	Vater et al. (2015)
968.7	Unknown fusaricidin [M+H]^+^	Vater et al. (2015)
982.7	Unknown fusaricidin [M+H]^+^	Vater et al. (2015)
*Paenibacillus* sp. RP31		
868.0	Fusaricidin A [M+H]^+^	Vater et al. (2015)
883.7	Fusaricidin A [M+H]^+^	Kajimura and Kaneda (1996); Vater et al. (2015)
897.4	Fusaricidin B [M+H]^+^	Kajimura and Kaneda (1997); Vater et al. (2015)
905.4	Fusaricidin A [M+Na]^+^	Vater et al. (2015)
919.4	Fusaricidin B [M+Na]^+^	Vater et al. (2015)
947.4	Fusaricidin C [M+H]^+^	Kajimura and Kaneda (1997); Vater et al. (2015)
961.4	Fusaricidin D [M+H]^+^	Kajimura and Kaneda (1997); Vater et al. (2015)
911.4	Fusaricidin A(C_17)_/E(C_15_) [M+H]^+^	Vater et al. (2017)
*P. kribbensis* CFCC 1865		
868.1	Fusaricidin A [M+H]^+^	Vater et al. (2015)
883.6	Fusaricidin A [M+H]^+^	Kajimura and Kaneda (1996); Vater et al. (2015)
897.6	Fusaricidin B [M+H]^+^	Kajimura and Kaneda (1997); Vater et al. (2015)
919.5	Fusaricidin B [M+Na]^+^	Vater et al. (2015)
911.6	Fusaricidin A(C_17)_/E(C_15_) [M+H]^+^	Vater et al. (2017)
925.6	Fusaricidin LI-F08b [M+H]^+^	Vater et al. (2015)
933.6	Fusaricidin LI-F05b [M+Na]^+^	Vater et al. (2015)
947.6	Fusaricidin C [M+H]^+^	Kajimura and Kaneda (1997); Vater et al. (2015)

The variety and quantity of the fusaricidins produced by the strains within the *P. polymyxa* complex are generally consistent with the extent of their antifungal activities. *P. peoriae* CGMCC 1.3761^*T*^ and CFCC 1854 produced fewer variety and amount of fusaricidins and showed weaker antifungal activities comparing with the other five strains. Therefore, fusaricidins are likely responsible for the antifungal activities against the broad-spectrum phytopathogenic fungi.

### Most Strains Within *Paenibacillus polymyxa* Complex Contain the *fus* Gene Cluster

In the whole genome sequences of 396 strains assigned to 165 *Paenibacillus* species and deposited in the NCBI database, the gene cluster for the biosynthesis of fusaricidins (*fus*) was detected from 88 strains (22% of 396 strains) using antiSMASH. The *fus* cluster appeared to be aggregated in closely related species. In particular, the *fus* cluster was aggregated in 73 (96%) of 76 strains within the *P. polymyxa* complex and in 8 of 10 strains within the *P. elgii* complex including *P. elgii*, *P. tyrfis*, and *P. tianmuensis* (Figure 1 and Supplementary Table 1). Gene clusters for biosynthesis of siderophores were detected from 99 strains (25% of 396 strains) and from 25 (33%) of 76 strains within the *P. polymyxa* complex (Supplementary Table 1). The genomes of nodule-inhabiting *P. lupini* type strain and *P. prosopidis* type strain out of the *P. polymyxa* complex (Figure 1) do not contain gene clusters for biosynthesis of fusaricidins and siderophores (Supplementary Table 1).

### Fusaricidin-Producing *Paenibacillus* Damaged Fungal Cell Structures

**FIGURE 4:**
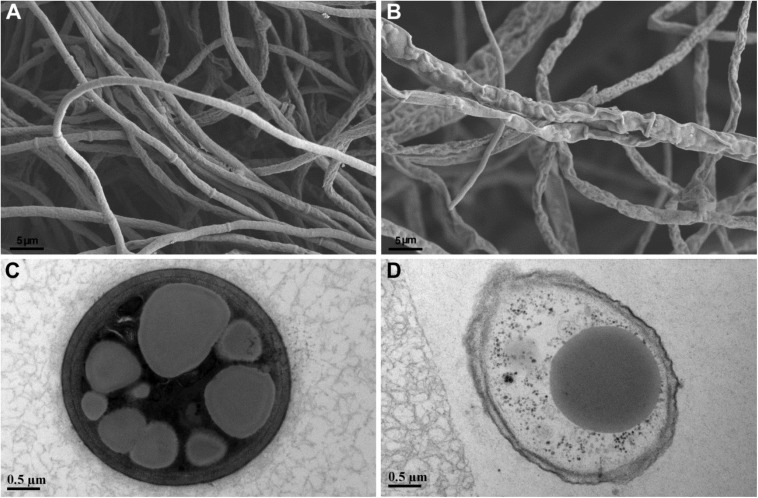
Structures of *Fusarium graminearum* hypha shown by scanning electron microscopy **(A,B)** and transmission electron microscopy **(C,D)**. Control hyphal cell walls are smooth **(A)** and intact and contain electron-dense cell contents and relatively electron-lucent lipid granules **(C)**. Hypha confronting *Paenibacillus peoriae* strain RP51 are shrunk and distorted **(B)**; incompletely developed cell walls are broken and in association with cellular vacuolization **(D)**.

*Paenibacillus peoriae* RP51 showing potent broad-spectrum antifungal activities and fusaricidin-producing activity was used to detect the antifungal action on fungal cell structures. Control *F. graminearum* hyphae grown on PDA were smooth and intact shown by SEM (Figure 4A); the hyphal cells contained electron-dense cell contents and electron-lucent lipid granules shown by TEM (Figure 4C). In contrast, the hypha of *F. graminearum* confronting RP51 were shrunk and distorted (Figure 4B); hyphal cell walls were broken while cell contents were leaked indicating by vacuolization (Figure 4D).

### Nodule-Inhabiting Strains Within *Paenibacillus polymyxa* Complex Can Colonize in Wheat Seedlings and Affect Plant Growth

**FIGURE 5:**
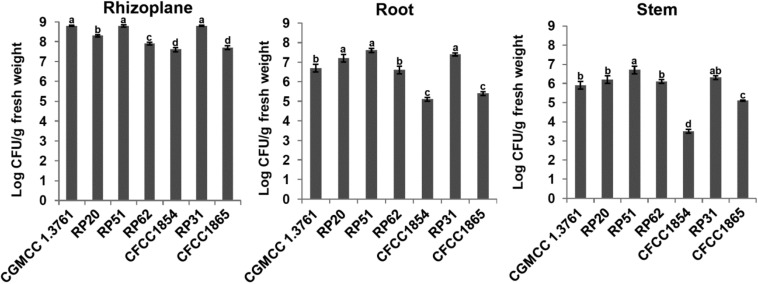
Colonization of wheat seedlings by *Paenibacillus peoriae* CGMCC 1.3761^*T*^ and nodule-inhabiting strains within *P. polymyxa* complex. Bacteria were recovered at 9 days after inoculation to imbibed wheat seeds. The different letters on the columns and standard error bars indicate significant difference between the treatments at *p* < 0.05.

Plant colonization by the nodule-inhabiting strains within the *P. polymyxa* complex was determined using wheat seedlings in a gnotobiotic condition. *P. peoriae* CGMCC 1.3761^*T*^ from soil was used for comparison. At 9 days after inoculation to imbibed wheat seeds, the *Paenibacillus* strains were recovered from rhizoplane and surface-sterilized roots and stems. The population of the nodule-inhabiting strains within the *P. polymyxa complex* at rhizoplane, in roots, and in stems was 4.0 × 10^7^ – 6.6 × 10^8^, 1.0 × 10^5^ – 4.0 × 10^7^, and 5.3 × 10^3^ – 5.0 × 10^6^ CFU g^–1^ fresh weight, respectively (Figure 5). Generally, *P. peoriae* RP51 and *Paenibacillus* sp. RP31 colonized at a relatively higher population level at rhizoplane, in roots, and in stems than those of the other four nodule-inhabiting strains (Figure 5). *P. peoriae* CGMCC 1.3761^*T*^ colonized at rhizoplane at a population level similar to that of strains RP51 and RP31 (Figure 5).

Strains within the *P. polymyxa* complex generally promoted seed germination except that *P. peoriae* CFCC 1854 inhibited seed germination (Table 4). At 9 days after inoculation, *P. peoriae* RP51 and *Paenibacillus* sp. RP31 significantly increased root length (48.5% and 49.7%), shoot length (26.7% and 29.6%), and dry weight (24.1% and 25.0%) of wheat seedlings; *P. peoriae* RP62 significantly increased dry weight (16.5%) of wheat seedlings; *P. peoriae* RP20 slightly increased root length, shoot length, and dry weight of wheat seedlings; *P. kribbensis* CFCC 1865 decreased dry weight of wheat seedlings; *P. peoriae* CFCC 1854 significantly decreased root length (67.8%), shoot length (38.0%), and dry weight (36.0%) of wheat seedlings. *P. peoriae* CGMCC 1.3761^*T*^ significantly increased the root length (41.5%) and dry weight (16.5%) of wheat seedlings (Table 4 and Supplementary Figure 4).

**TABLE 4:** Growth parameters of wheat seedlings inoculated with strains within *Paenibacillus polymyxa* complex.

**Treatments**	Seed germination (%)*	Root length (mm)*	Shoot length (mm)*	Dry weight (mg)*
Control without inoculation	77.8 ± 2.2^*b*^	72.6 ± 6.2^*c*^	70.0 ± 5.0^*b*^	22.8 ± 0.9^*c*^
*P. peoriae* CGMCC 1.3761^*T*^	88.9 ± 2.2^*a*^	102.7 ± 6.7^*ab*^	85.0 ± 3.6^*ab*^	27.3 ± 0.6^*ab*^
*P. peoriae* RP20	80.0 ± 3.9^*a*^	78.0 ± 5.2^*cd*^	76.3 ± 7.8^*ab*^	25.1 ± 0.7^*bc*^
*P. peoriae* RP51	88.9 ± 4.4^*a*^	107.8 ± 8.8^*a*^	88.7 ± 2.5^*a*^	28.3 ± 0.4^*a*^
*P. peoriae* RP62	84.4 ± 5.9^*a*^	96.4 ± 8.6^*abc*^	80.0 ± 4.8^*ab*^	27.3 ± 0.8^*ab*^
*P. peoriae* CFCC 1854	66.6 ± 6.7^*b*^	23.4 ± 5.6^*e*^	43.4 ± 7.4^*c*^	14.6 ± 0.9^*e*^
*Paenibacillus* sp. RP31	84.5 ± 2.2^*a*^	108.7 ± 3.4^*a*^	90.7 ± 2.2^*a*^	28.5 ± 0.3^*a*^
*P. kribbensis* CFCC 1865	82.2 ± 2.2^*a*^	84.8 ± 5.6^*bcd*^	69.9 ± 2.4^*b*^	20.1 ± 0.8^*d*^

*Data are presented as mean value ± standard error. The different letters following the values in the same column indicate significant difference between the treatments at *p* < 0.05.

### Nodule-Inhabiting Strains Within *Paenibacillus polymyxa* Complex Can Produce IAA and Dissolve Phosphate or Fix N_2_

**FIGURE 6:**
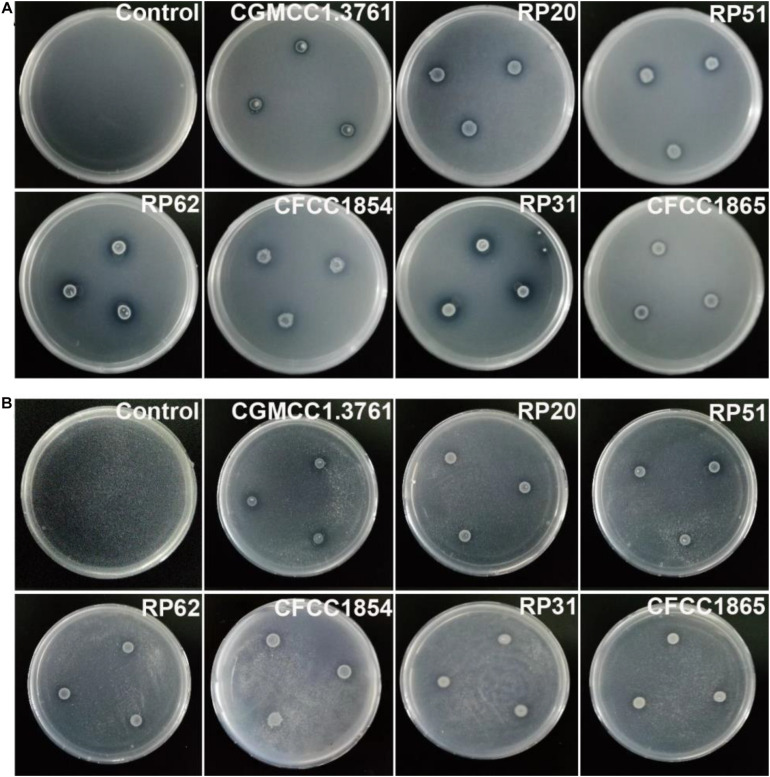
Agar plate assays show solubilization of calcium phosphate **(A)** and calcium phytate **(B)** around colonies of *P. peoriae* CGMCC 1.3761^*T*^ and nodule-inhabiting strains within *Paenibacillus polymyxa* complex.

**FIGURE 7:**
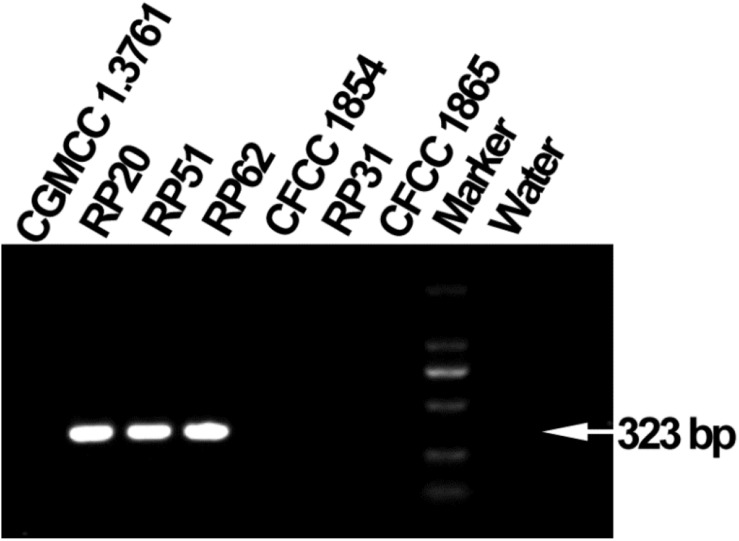
Agarose gel electrophoresis shows PCR amplification of *nifH* gene fragments (323 bp) from *P. peoriae* strains RP20, RP51, and RP62 but not *P. peoriae* strain CGMCC 1.3761^*T*^ and CFCC 1854, *Paenibacillus* sp. RP31, and *P. kribbensis* CFCC1865.

The six nodule-inhabiting strains within the *P. polymyxa complex* and *P. peoriae* CGMCC 1.3761^*T*^ from soil were able to produce IAA about 5 μg from cultures at 1 × 10^9^ CFU ml^–1^. They were also able to dissolve calcium phosphate rich in alkaline soils, and calcium phytate in soils rich in organic phosphate (Figure 6) but not ferric phosphate rich in acidic soils. *P. peoriae* RP20, RP51, and RP62 were positive for *nifH* gene amplification (Figure 7) and could grow well on the nitrogen-free Jensen’s agar medium. These nodule-inhabiting strains may stimulate the seed germination and seedling growth by producing IAA. *P. peoriae* RP20, RP51, and RP62 may fix N_2_ in association with seedlings in the gnotobiotic culture without external nutrient supply but their phosphate solubilization activity was not involved in their plant growth promotion in the gnotobiotic culture without insoluble phosphates.

### All *Paenibacillus* Strains Contain Genes for Biosynthesis of IAA and Nearly Half Within *Paenibacillus polymyxa* Complex Contain Nitrogenase Genes

In the whole genome sequences of 396 strains belonging to 165 *Paenibacillus* species, all contain the *ipdC* (=*ilvB*) gene encoding the key enzyme (indole-3-pyruvate decarboxylase = biosynthetic-type acetolactate synthase large subunit) for biosynthesis of IAA via the IPyA pathway; 109 strains (28% of 396 strains) and 33 (43%) of 76 strains within the *P. polymyxa* complex (Supplementary Table 1) contain nitrogenase genes. Among 5216 *Bacillus* strains whose whole genome sequences were deposited in the NCBI database, only five strains assigned to the genus *Bacillus* (*Bacillus caseinilyticus* SP^*T*^, *B. nealsonii* FO-92^*T*^, *Bacillus* sp. 03113, *Bacillus* sp. 522_BSPC and MB2021) contain the minimal nitrogen fixation gene (*nif*) cluster (Wang et al., 2013) and their nitrogenase amino acid sequences are most closely related to those of *Paenibacillus*, such as their nitrogenase iron proteins (NifH) have identities of 86–89% to those of *Paenibacillus*. In addition, all the 396 *Paenibacillus* genomes and 5216 *Bacillus* genomes do not contain genes encoding ACC deaminase.

### *P. peoriae* RP51 and *Paenibacillus* sp. RP31 Can Control Fungal Infection *in planta*

*Paenibacillus peoriae* RP51 and *Paenibacillus* sp. RP31 showed potent antifungal activities *in vitro*, potent plant colonization and plant growth-promoting competence. They were selected to evaluate their potential in biocontrol of fungal diseases *in planta* using the pathosystem of FRR and FFR on wheat seedlings (Colombo et al., 2019). After inoculation of a Fusarium plug on a primary root at 10 mm distance from the seed, necrosis of plant tissues occurred along the roots (root rot) and shoots (foot rot) of wheat seedlings (Supplementary Figure 1). At 4 days after fungal inoculation, strain RP51 and strain RP31, which were inoculated to wheat seeds, inhibited the FRR symptom about 66% and 60%, respectively (Table 5). At 6 days after fungal inoculation, strain RP51 and strain RP31 inhibited the FFR symptom about 61% and 55%, respectively (Table 5).

**TABLE 5:** Fusarium root rot (FRR) and Fusarium foot rot (FFR) in wheat seedlings controlled by *Paenibacillus peoriae* RP51 and *Paenibacillus* sp. RP31.

**Treatment**	FRR length (mm)*	Inhibition of FRR (%)*	FFR severity (%)*	Inhibition of FFR (%)*
Sterile PDA plug	0	No FRR	0	No FFR
*F. graminearum*	8.9 ± 0.3^*a*^	0	40.8 ± 0.8^*a*^	0
*F. graminearum* and RP51	3.1 ± 0.0^*b*^	65.6 ± 0.4^*a*^	15.8 ± 0.8^*b*^	61.2 ± 2.0^*a*^
*F. graminearum* and RP31	3.5 ± 0.1^*c*^	60.3 ± 1.0^*b*^	18.3 ± 0.8^*c*^	55.1 ± 2.0^*b*^

*Data are presented as mean value ± standard error. The different letters following the values in the same column indicate significant difference between the treatments at *p* < 0.05.

## Discussion

The genus *Paenibacillus* is a ubiquitously occurring bacterial genus and comprises over 250 species^3^. *Paenibacillus* is known for its activities against phytopathogens and for plant growth promotion (Grady et al., 2016; Rybakova et al., 2016). Many *Paenibacillus* isolates have been found in legume root nodules (De Meyer et al., 2015; Pandya et al., 2015). Few studies have explored the functions of nodule-inhabiting *Paenibacillus* for plant hosts (e.g., Ferchichi et al., 2020) and biocontrol of a certain plant pathogen and disease (Senthilkumar et al., 2007). Our study explores nodule-inhabiting *Paenibacillus*’ antifungal activities and biocontrol potentials against broad-spectrum important phytopathogenic fungi.

Our phylogenetic analysis and previous phylogenetic analyses (Rybakova et al., 2016; Wang et al., 2018; Kwak et al., 2020; Velazquez et al., 2020) based on 16S rRNA gene sequences show that *P. polymyxa*, *P. peoriae*, *P. kribbensis*, *P. ottowii*, *P. brasilensis*, *P. terrae*, and “*P. maysiensis*” (not validly published; Wang et al., 2018) formed a monophyletic complex (*Paenibacillus polymyxa* complex). We screened out 6 *Paenibacillus* strains showing broad-spectrum antagonistic activities against five important phytopathogenic fungi from 11 nodule-inhabiting strains belonging to seven to eight *Paenibacillus* species. The six *Paenibacillus* strains against broad-spectrum phytopathogenic fungi consist of four *P. peoriae* strains (RP20, RP51, RP62, and CFCC 1854), one *P. kribbensis* strain CFCC1865 and one strain RP31 belonging to *P. kribbensis* or a novel species closely related to *P. kribbensis*. A previously reported nodule-inhabiting strain HKA-15 showing antagonistic activities against broad-spectrum phytopathogenic fungi and bacteria (Senthilkumar et al., 2009; Annapurna et al., 2013) may belong to the same species as strain RP31. Interestingly, all the known nodule-inhabiting *Paenibacillus* strains against broad-spectrum phytopathogenic fungi belong to the monophyletic *P. polymyxa* complex (Figure 1).

Our MALDI-TOF-MS analysis identified multiple fusaricidins produced by the nodule-inhabiting strains within the *P. polymyxa* complex. The variety and quantity of the fusaricidins produced by these strains within the *P. polymyxa* complex are generally consistent with the extent of their antifungal activities. Likely, fusaricidins are responsible for the antifungal activities against broad-spectrum phytopathogenic fungi by the nodule-inhabiting strains within the *P. polymyxa* complex.

Fusaricidins and the single *fus* operon encoding a nonribosomal peptide synthetase (*fusA*) for the biosynthesis of a variety of fusaricidins (Han et al., 2012) have been demonstrated to play roles in *P. polymyxa* against broad-spectrum fungal pathogens (Choi et al., 2008; Li et al., 2013; Li and Chen, 2019). Fusaricidins produced by *P. polymyxa* have been demonstrated to inhibit *Fusarium* spore germination and disrupt hyphal membranes (Li and Chen, 2019). Likely, fusaricidins are responsible for the damage of *Fusarium* hyphal cells by *P. peoriae* RP51 found in this study. Notably, our genome mining revealed that 83% (73 of 88) *fus*-containing strains in the genus *Paenibacillus* belong to the *P. polymyxa* complex and 96% (73 of 76) strains within the *P. polymyxa* complex contain the *fus* cluster. Consistently, Liu et al. (2019) showed that all isolated nitrogen-fixing strains within the *P. polymyxa* complex (*n* = 20) inhibited the growth of four or over four of six tested phytopathogenic fungi whereas other five strains outside of the *P. polymyxa* complex did not inhibit any of the six tested fungi. Fusaricidins produced by *P. polymyxa* have also been demonstrated to induce plant systemic resistance via salicylic acid pathway against *Fusarium* and *Phytophthora* pathogens (Lee et al., 2013; Li and Chen, 2019). Therefore, the *P. polymyxa* complex is a promising resource for fusaricidin-dependent control of broad-spectrum fungal pathogens and inducing plant systemic resistance.

Our genome mining also revealed that all strains (*n* = 76) within the *P. polymyxa* complex contain the *ilvB* (=*ipdC*) gene and 43% of them (33 of 76) contain the *nif* cluster. Consistently, the six nodule-inhabiting strains within the *P. polymyxa* complex can produce IAA and half can fix N_2_. Likewise, Quyet-Tien et al. (2010) found all tested *P. polymyxa* strains (*n* = 29) producing IAA and 38% of them (11 of 29) containing the *nifH* gene. Liu et al. (2019) showed that almost all nitrogen-fixing strains (19 of 20) within the *P. polymyxa* complex produced IAA. The nodule-inhabiting strain HKA-15 within the *P. polymyxa* complex also contains the *nifH* gene (Senthilkumar et al., 2009). Anand et al. (2013) and Padda et al. (2016) did ^15^N dilution assay and revealed that *P. polymyxa* promoted plant growth via nitrogen fixation. In contrast, only five of 5216 strains assigned to the genus *Bacillus* contain the *nif* clusters, which are most closely related to those of *Paenibacillus*. Most likely, nitrogen-fixing strains within the *P. polymyxa* complex have the advantage of nitrogen fixation over *Bacillus* strains as biocontrol agents or biofertilizers.

Together, production of fusaricidins and IAA and nitrogen fixation are likely conserved in the *P. polymyxa* complex and evolved in their adaptations to the plant-associated life (Xie et al., 2016; Wang et al., 2020; Zhou et al., 2020). Our genome mining and screening of *Paenibacillus* strains revealed that the strains within the *P. polymyxa* complex have a high probability to be plant growth promoters and biocontrol agents against broad-spectrum phytopathogenic fungi. However, there are exceptions for the strains within the *P. polymyxa* complex to have broad-spectrum antifungal activities (e.g., *P. peoriae* type strain) or promote plant growth (e.g., *P. peoriae* CFCC 1854). Therefore, screening of the strains within the *P. polymyxa* complex for biocontrol and biofertilization is necessary. We screened out *P. peoriae* RP51 and *Paenibacillus* sp. RP31 showing broad-spectrum antifungal activities, potent plant colonization competence and plant growth-promoting activities, and effective *in planta* control of fungal infection.

Moreover, we propose an effective approach to screen *Paenibacillus* strains to be effective biocontrol agents and biofertilizers based on our study and other studies (e. g. Quyet-Tien et al., 2010; Colombo et al., 2019; Liu et al., 2019). First, phylogenetic analysis of nearly complete 16S rRNA gene sequences identifies strains within the *P. polymyxa* complex, which most likely produce fusaricidins and IAA. Second, confrontation culture of *Paenibacillus* and pathogens and amplification of *nifH* gene screen out *Paenibacillus* strains having broad-spectrum antifungal activity and nitrogen-fixing ability. Third, gnotobiotic cultures of plant seedlings, *Paenibacillus*, and pathogens screen out *Paenibacillus* strains having plant colonization competence and *in planta* biocontrol potential. Fourth, field tests on the promising *Paenibacillus* strains, such as *P. peoriae* RP51, determine effective biocontrol agents and biofertilizers, leading to reduced use of agrochemicals for sustainable agriculture.

Conclusively, our study highlights that endophytic strains within the *P. polymyxa* complex have a high probability to be effective biocontrol agents and biofertilizers.

## Data Availability Statement

The dataset (Supplementary Table 1) generated for this study can be found in the online repository. The nucleotide sequences generated in this study were deposited at: https://www.ncbi.nlm.nih.gov/genbank/ under the accession numbers MN715870, MN715872, MN715875, MT093458, MN715871, MT093459, MT093460, MT093461, and MT040706.

## Author Contributions

MA, LL, XL, YY, and QA contributed to the conceptualization. LL, YY, and QA contributed to the microbial resources. MA, YL, RH, AH, and TX contributed to the investigation. MA and QA contributed to the data analysis. MA and QA contributed to the manuscript preparation. XL, BL, JY, and QA contributed to the review and editing. BL, JY, and QA contributed to the supervision. LL, BL, JY, and QA contributed to the funding acquisition. All authors contributed to the article and approved the submitted version.

## Conflict of Interest

The authors declare that the research was conducted in the absence of any commercial or financial relationships that could be construed as a potential conflict of interest.

## Footnotes

1 www.ezbiocloud.net

2 www.ncbi.nlm.nih.gov/genome/

3 https://lpsn.dsmz.de/genus/paenibacillus

## References

[B1] Ali M. A. Ren H. Ahmed T. Luo J. An Q. Qi X. (2020). Antifungal effects of rhizospheric bacillus species against bayberry twig blight pathogen *Pestalotiopsis versicolor*. *Agronomy* 10 1811 10.3390/agronomy10111811

[B2] Alori E. T. Dare M. O. Babalola O. O. (2017). “Microbial inoculants for soil quality and plant health,” in *Sustainable Agriculture Reviews*, Vol. 22 ed. Lichtfouse E., (Cham: Springer), 281–307. 10.1007/978-3-319-48006-0_9

[B3] Anand R. Grayston S. Chanway C. (2013). N2-fixation and seedling growth promotion of lodgepole pine by endophytic *Paenibacillus polymyxa*. *Microb. Ecol.* 66 369–374. 10.1007/s00248-013-0196-1 23420205

[B4] Annapurna K. Ramadoss D. Bose P. Vithal K. L. (2013). In situ localization of *Paenibacillus polymyxa* HKA-15 in roots and root nodules of soybean (*Glycine max*. L.). *Plant Soil* 373 641–648. 10.1007/s11104-013-1825-7

[B5] Bashan Y. Kamnev A. A. de-Bashan L. E. (2013). Tricalcium phosphate is inappropriate as a universal selection factor for isolating and testing phosphate-solubilizing bacteria that enhance plant growth: a proposal for an alternative procedure. *Biol. Fertil. Soils* 49 465–479. 10.1007/s00374-012-0737-7

[B6] Blin K. Shaw S. Steinke K. Villebro R. Ziemert N. Lee S. Y. (2019). antiSMASH 5.0: updates to the secondary metabolite genome mining pipeline. *Nucleic Acids Res.* 47 W81–W87. 10.1093/nar/gkz310 31032519 PMC6602434

[B7] Carro L. Flores-Felix J. D. Cerda-Castillo E. Ramirez-Bahena M. H. Igual J. M. Tejedor C. (2013). *Paenibacillus endophyticus* sp. nov., isolated from nodules of Cicer arietinum. *Int. J. Syst. Evol. Microbiol.* 63 4433–4438. 10.1099/ijs.0.050310-0 23852155

[B8] Carro L. Flores-Felix J. D. Ramirez-Bahena M. H. Garcia-Fraile P. Martinez-Hidalgo P. Igual J. M. (2014). *Paenibacillus lupini* sp. nov., isolated from nodules of Lupinus albus. *Int. J. Syst. Evol. Microbiol.* 64 3028–3033. 10.1099/ijs.0.060830-0 24928428

[B9] Carvalho F. P. (2017). Pesticides, environment, and food safety. *Food Energy Sec.* 6 48–60. 10.1002/fes3.108

[B10] Cassán F. Coniglio A. López G. Molina R. Nievas S. de Carlan C. L. N. (2020). Everything you must know about *Azospirillum* and its impact on agriculture and beyond. *Biol. Fertil. Soils* 56 461–479. 10.1007/s00374-020-01463-y

[B11] Choi S. K. Park S.-Y. Kim R. Lee C. H. Kim J. F. Park S. H. (2008). Identification and functional analysis of the fusaricidin biosynthetic gene of *Paenibacillus polymyxa* E681. *Biochem. Biophys. Res. Commun.* 365 89–95. 10.1016/j.bbrc.2007.10.147 17980146

[B12] Colombo E. M. Kunova A. Pizzatti C. Saracchi M. Cortesi P. Pasquali M. (2019). Selection of an endophytic *Streptomyces* sp. strain DEF09fromwheat roots as a biocontrol agent against Fusarium graminearum. *Front. Microbiol.* 10:2356. 10.3389/fmicb.2019.02356 31681219 PMC6798073

[B13] De Meyer S. E. De Beuf K. Vekeman B. Willems A. (2015). A large diversity of non-rhizobial endophytes found in legume root nodules in Flanders (Belgium). *Soil Biol. Biochem.* 83 1–11. 10.1016/j.soilbio.2015.01.002

[B14] Ding Y. Wang J. Liu Y. Chen S. (2005). Isolation and identification of nitrogen-fixing bacilli from plant rhizospheres in Beijing region. *J. Appl. Microbiol.* 99 1271–1281. 10.1111/j.1365-2672.2005.02738.x 16238759

[B15] Ferchichi N. Toukabri W. Boularess M. Smaoui A. Mhamdi R. Trabelsi D. (2019). Isolation, identification and plant growth promotion ability of endophytic bacteria associated with lupine root nodule grown in Tunisian soil. *Arch. Microbiol.* 201 1333–1349. 10.1007/s00203-019-01702-3 31309236

[B16] Ferchichi N. Toukabri W. Vrhovsek U. Angeli A. Masuero D. Mhamdi R. (2020). Inoculation of Lupinus albus with the nodule-endophyte *Paenibacillus glycanilyticus* LJ121 improves grain nutritional quality. *Arch. Microbiol.* 202 283–291. 10.1007/s00203-019-01745-6 31650197

[B17] Gardener B. B. M. (2004). Ecology of Bacillus and *Paenibacillus* spp. in agricultural systems. *Phytopathology* 94 1252–1258. 10.1094/PHYTO.2004.94.11.1252 18944463

[B18] Grady E. N. MacDonald J. Liu L. Richman A. Yuan Z. C. (2016). Current knowledge and perspectives of *Paenibacillus*: a review. *Microb. Cell Fact.* 15 203. 10.1186/s12934-016-0603-7 27905924 PMC5134293

[B19] Hallman J. Quadt-Hallman A. Mahaffee W. Kloepper J. (1997). Bacterial endophytes in agricultural crops. *Can. J. Microbiol.* 43 895–914. 10.1139/m97-131

[B20] Han J. W. Kim E. Y. Lee J. M. Kim Y. S. Bang E. Kim B. S. (2012). Site-directed modification of the adenylation domain of the fusaricidin nonribosomal peptide synthetase for enhanced production of fusaricidin analogs. *Biotechnol. Lett.* 34 1327–1334. 10.1007/s10529-012-0913-8 22450515

[B21] Hardoim P. R. van Overbeek L. S. Berg G. Pirttilä A. M. Compan S. Campisano A. (2015). The hidden world within plants: ecological and evolutionary considerations for defining functioning of microbial endophytes. *Microbiol. Mol. Biol. Rev.* 79 293–320. 10.1128/MMBR.00050-14 26136581 PMC4488371

[B22] Kajimura Y. Kaneda M. (1996). Fusaricidin A, a new depsipeptide antibiotic produced by Bacillus polymyxa KT-8. Taxonomy, fermentation, isolation, structure elucidation and biological activity. *J. Antibiot (Tokyo)* 49 129–135. 10.7164/antibiotics.49.129 8621351

[B23] Kajimura Y. Kaneda M. (1997). Fusaricidins B, C and D, new depsipeptide antibiotics produced by Bacillus polymyxa KT-8: isolation, structure elucidation and biological activity. *J Antibiot (Tokyo)* 50 220–228. 9439693

[B24] Kwak M. J. Choi S. B. Ha S. M. Kim E. H. Kim B. Y. Chun J. (2020). Genome-based reclassification of Paenibacillus jamilae Aguilera et al. 2001 as a later heterotypic synonym of Paenibacillus polymyxa (Prazmowski 1880) Ash et al. 1994. *Int. J. Syst. Evol. Microbiol.* 70 3134–3138. 10.1099/ijsem.0.004140 32375953

[B25] Lai W. A. Hameed A. Lin S. Y. Hung M. H. Hsu Y. H. Liu Y. C. (2015). Paenibacillus medicaginis sp. nov. achitinolytic endophyte isolated from a root nodule of alfalfa (*Medicago sativa* L.). *Int. J. Syst. Evol. Microbiol.* 65 3853–3860. 10.1099/ijsem.0.000505 28875919

[B26] Lamichhane J. R. Dachbrodt-Saaydeh S. Kudsk P. Messéan A. (2016). Toward a reduced reliance on conventional pesticides in European agriculture. *Plant Dis.* 100 10–24. 10.1094/PDIS-05-15-0574-FE 30688570

[B27] Lee S. H. Cho Y. E. Park S. H. Balaraju K. Park J. W. Lee S. W. (2013). An antibiotic fusaricidin: a cyclic depsipeptide from *Paenibacillus polymyxa* E681 induces systemic resistance against Phytophthora blight of red-pepper. *Phytoparasitica* 41 49–58. 10.1007/s12600-012-0263-z

[B28] Li S. Zhang R. Wang Y. Zhang N. Shao J. Qiu M. (2013). Promoter analysis and transcription regulation of fus gene cluster responsible for fusaricidin synthesis of *Paenibacillus polymyxa* SQR-21. *Appl. Microbiol. Biotechnol.* 97 9479–9489. 10.1007/s00253-013-5157-6 24072159

[B29] Li Y. Chen S. (2019). Fusaricidin produced by *Paenibacillus polymyxa* WLY78 induces systemic resistance against Fusarium wilt of cucumber. *Int. J. Mol. Sci.* 20 5240. 10.3390/ijms20205240 31652608 PMC6829208

[B30] Liu H. Tian W. Li B. Wu G. Ibrahim M. Tao Z. (2012). Antifungal effect and mechanism of chitosan against the rice sheath blight pathogen, *Rhizoctonia solani*. *Biotechnol. Lett.* 34 2291–2298. 10.1007/s10529-012-1035-z 22932934

[B31] Liu X. Li Q. Li Y. Guan G. Chen S. (2019). Paenibacillus strains with nitrogen fixation and multiple beneficial properties for promoting plant growth. *PeerJ.* 7 e7445. 10.7717/peerj.7445 31579563 PMC6761918

[B32] Mahasneh A. M. Stewart D. J. (1980). A medium for detecting β-(1→3) glucanase activity in bacteria. *J. Appl. Bacteriol.* 48 457–458. 10.1111/j.1365-2672.1980.tb01035.x

[B33] Masum M. M. I. Liu L. Yang M. Hossain M. M. Siddiqa M. M. Supty M. M. (2018). Halotolerant bacteria belonging to operational group Bacillus amyloliquefaciens in biocontrol of the rice brown stripe pathogen *Acidovorax oryzae*. *J. Appl. Microbiol.* 125 1852–1867. 10.1111/jam.14088 30146698

[B34] Menéndez E. Ramírez-Bahena M. H. Carro L. Fernández-Pascual M. Peter Klenk H. Velázquez E. (2016). Paenibacillus periandrae sp. nov., isolated from nodules of *Periandra mediterranea*. *Int. J. Syst. Evol. Microbiol.* 66 1838–1843. 10.1099/ijsem.0.000953 26843192

[B35] Nautiyal C. S. (1999). An efficient microbiological growth medium for screening phosphate solubilizing microorganisms. *FEMS Microbiol. Lett.* 170 265–270. 10.1111/j.1574-6968.1999.tb13383.x 9919677

[B36] Padda K. P. Puri A. Chanway C. P. (2016). Effect of GFP tagging of *Paenibacillus polymyxa* P2b-2R on its ability to promote growth of canola and tomato seedlings. *Biol Fertil Soils* 52 377–387. 10.1007/s00374-015-1083-3

[B37] Pandya M. Rajput M. Rajkumar S. (2015). Exploring plant growth promoting potential of non rhizobial root nodules endophytes of *Vigna radiata*. *Microbiology* 84 80–89. 10.1134/S0026261715010105

[B38] Que Y. Yue X. Yang N. Xu Z. Tang S. Wang C. (2020). Leucine biosynthesis is required for infection-related morphogenesis and pathogenicity in the rice blast fungus *Magnaporthe oryzae*. *Curr. Genet.* 66 155–171. 10.1007/s00294-019-01009-2 31263943

[B39] Quyet-Tien P. Park Y. M. Seul K. J. Ryu C. M. Park S. H. Kim J. G. (2010). Assessment of root-associated *Paenibacillus polymyxa* groups on growth promotion and induced systemic resistance in pepper. *J. Microbiol. Biotechnol.* 20 1605–1613. 10.4014/JMB.1007.07014 21193814

[B40] Riungu G. M. Muthorni J. W. Narla R. D. Wagacha J. M. Gathumbi J. K. (2008). Management of Fusarium head blight of wheat and deoxynivalenol accumulation using antagonistic microorganisms. *Plant Pathol. J.* 7 13–19. 10.3923/ppj.2008.13.19

[B41] Rosenblueth M. Martínez-Romero E. (2006). Bacterial endophytes and their interactions with hosts. *Mol. Plant Microbe Interact.* 19 827–837. 10.1094/MPMI-19-0827 16903349

[B42] Rybakova D. Cernava T. Köberl M. Liebminger S. Etemadi M. Berg G. (2016). Endophytes-assisted biocontrol: novel insights in ecology and the mode of action of *Paenibacillus*. *Plant Soil* 405 125–140. 10.1007/s11104-015-2526-1

[B43] Sarwar M. Kremer R. J. (1995). Determination of bacterially derived auxins using a microplate method. *Lett. Appl. Microbiol.* 20 282–285. 10.1111/j.1472-765X.1995.tb00446.x

[B44] Schwyn B. Neilands J. B. (1987). Universal chemical assay for the detection and determination of siderophores. *Anal. Biochem.* 160 47–56. 10.1016/0003-2697(87)90612-9 2952030

[B45] Senthilkumar M. Govindasamy V. Annapurna K. (2007). Role of antibiosis in suppression of charcoal rot disease by soybean endophyte Paenibacillus sp. HKA-15. *Curr. Microbiol.* 55 25–29. 10.1007/s00284-006-0500-0 17554471

[B46] Senthilkumar M. Swarnalakshmi K. Govindasamy V. Lee Y. K. Annapurna K. (2009). Biocontrol potential of soybean bacterial endophytes against charcoal rot fungus, *Rhizoctonia bataticola*. *Curr. Microbiol.* 58 288–293. 10.1007/s00284-008-9329-z 19067044

[B47] Shao W. Zhang Y. Wang J. Lv C. Chen C. (2016). BcMtg2 is required for multiple stress tolerance, vegetative development and virulence in *Botrytis cinerea*. *Sci. Rep.* 6 28673. 10.1038/srep28673 27346661 PMC4921815

[B48] Souza C. P. Burbano-Rosero E. M. Almeida B. C. Martins G. G. Albertini L. S. Rivera I. N. G. (2009). Culture medium for isolating chitinolytic bacteria from seawater and plankton. *World J. Microbiol. Biotechnol.* 25 2079–2082. 10.1007/s11274-009-0098-z

[B49] Tamura K. Peterson D. Peterson N. Stecher G. Nei M. Kumar S. (2011). MEGA5:molecular evolutionary genetics analysis using maximum likelihood, evolutionary distance, and maximum parsimony methods. *Mol. Biol. Evol.* 28 2731–2739. 10.1093/molbev/msr121 21546353 PMC3203626

[B50] Valverde A. Fterich A. Mahdhi M. Ramirez-Bahena M. H. Caviedes M. A. Mars M. (2010). Paenibacillus prosopidis sp. nov., isolated from the nodules of *Prosopis farcta*. *Int. J. Syst. Evol. Microbiol.* 60 2182–2186. 10.1099/ijs.0.014241-0 19897617

[B51] Vater J. Herfort S. Doellinger J. Weydmann M. Dietel K. Faetke S. (2017). Fusaricidins from Paenibacillus polymyxa M-1, a family of lipohexapeptides of unusual complexity-a mass spectrometric study. *J. Mass Spectrom* 52 7–15. 10.1002/jms.3891 27714901

[B52] Vater J. Niu B. Dietel K. Borriss R. (2015). Characterization of novel fusaricidins produced by Paenibacillus polymyxa-M1 using MALDI-TOF mass spectrometry. *J. Am. Soc. Mass Spectrom* 26 1548–1558. 10.1007/s13361-015-1130-1 26100395

[B53] Vater J. Xuewen G. Gabriele H. Christopher W. Peter F. (2003). ”Whole cell” matrix-assisted laser desorption ionization-time of flight-nass spectrometry, an emerging technique for efficient screening of biocombinatorial libraries of natural compounds -present state of research. *Comb. Chem.* 6 557–567. 10.2174/138620703106298725 14529380

[B54] Velazquez L. F. Rajbanshi S. Guan S. Hinchee M. Welsh A. (2020). Paenibacillus ottowii sp. nov. isolated from a fermentation system processing bovine manure. *Int. J. Syst. Evol. Microbiol.* 70 1463–1469. 10.1099/ijsem.0.003672 31961287

[B55] Wang B. Cheng H. Qian W. Zhao W. Liang C. Liu C. (2020). Comparative genome analysis and mining of secondary metabolites of *Paenibacillus polymyxa*. *Genes Genet. Syst.* 95 141–150. 10.1266/ggs.19-00053 32611933

[B56] Wang L. Zhang L. Liu Z. Zhao D. Liu X. Zhang B. (2013). A minimal nitrogen fixation gene cluster from Paenibacillus sp. WLY78 enables expression of active nitrogenase in *Escherichia coli*. *PLoS Genet.* 9:e1003865. 10.1371/journal.pgen.1003865 24146630 PMC3798268

[B57] Wang T. S. Xie J. Y. Wang L. Y. Chen S. F. (2018). Paenibacillus maysiensis sp. nov., a nitrogen-fixing species isolated from the rhizosphere soil of maize. *Curr. Microbiol.* 75 1267–1273. 10.1007/s00284-018-1519-8 29948008

[B58] Wilson D. (1995). Endophyte: the evolution of a term, and clarification of its use and definition. *Oikos* 73 274–276. 10.2307/3545919]

[B59] Xie J. Shi H. Du Z. Wang T. Liu X. Chen S. (2016). Comparative genomic and functional analysis reveal conservation of plant growth promoting traits in Paenibacillus polymyxa and its closely related species. *Sci. Rep.* 6 21329. 10.1038/srep21329 26856413 PMC4746698

[B60] Xie J. B. Du Z. Bai L. Tian C. Zhang Y. Xie J. Y. (2014). Comparative genomic analysis of N2-fixing and non-N2-fixing *Paenibacillus* spp.: organization, evolution and expression of the nitrogen fixation genes. *PLoS Genet.* 10:e1004231. 10.1371/journal.pgen.1004231 24651173 PMC3961195

[B61] Xu T. Yao F. Liang W.-S. Li Y.-H. Li D.-R. Wang H. (2012). Involvement of alternative oxidase in the regulation of growth, development, and resistance to oxidative stress of *Sclerotinia sclerotiorum*. *J. Microbiol.* 50 594–602. 10.1007/s12275-012-2015-7 22923107

[B62] Yin J. He D. Li X. Zeng X. Tian M. Cheng G. (2015). *Paenibacillus enshidis* sp. nov., isolated from the nodules of *Robinia pseudoacacia*. L. *Curr. Microbiol.* 71 321–325. 10.1007/s00284-015-0854-2 26063444

[B63] Yin Y. Wang Z. Cheng D. Chen X. Chen Y. Ma Z. (2018). The ATP-binding protein FgArb1 is essential for penetration, infectious and normal growth of *Fusarium graminearum*. *New Phytol.* 219 1447–1466. 10.1111/nph.15261 29932228

[B64] Zhou L. Zhang T. Tang S. Fu X. Yu S. (2020). Pan-genome analysis of *Paenibacillus polymyxa* strains reveals the mechanism of plant growth promotion and biocontrol. *Antonie Leeuwenhoek* 113 1539–1558. 10.1007/s10482-020-01461-y 32816227

