# Supplementary material for: Functional Analysis and Genome Mining Reveal High Potential of Biocontrol and Plant Growth Promotion in Nodule-Inhabiting Bacteria Within Paenibacillus polymyxa Complex
Source: Front Microbiol. 2021 Jan 18;11:618601. doi: 10.3389/fmicb.2020.618601 (PMC7848036; doi:10.3389/fmicb.2020.618601)
Supplement: Supplementary file 2 [file Data_Sheet_1.pdf]

Supplementary Material

Supplementary Figures

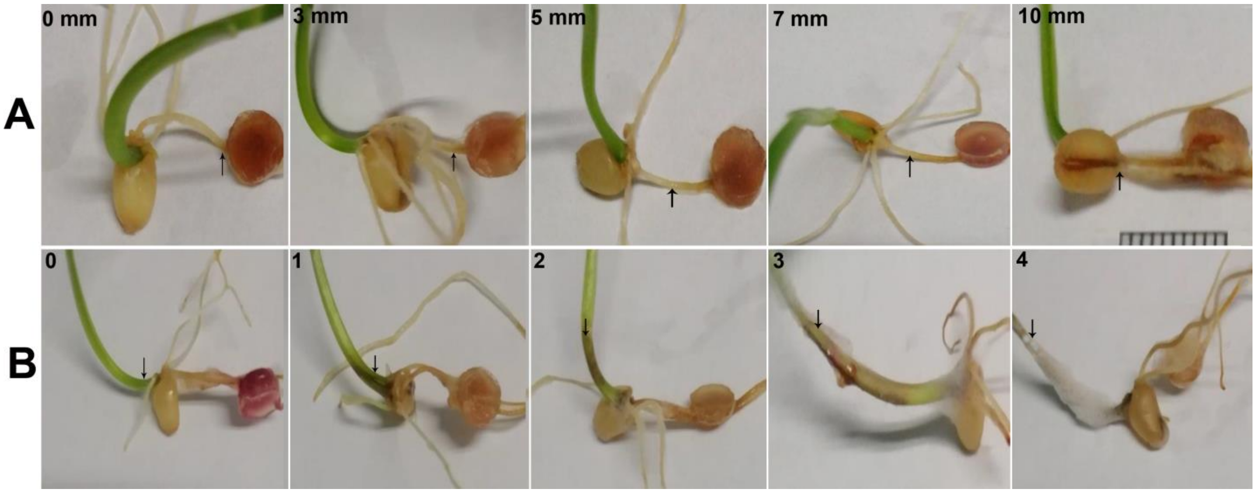

Supplementary Figure 1 Methods for assessing Fusarium root rot (FRR) and Fusarium foot rot (FFR) symptoms on wheat seedlings. (A) Length of necrosis of FRR measured from the inoculation point (0, 3, 5, 7, or 10 mm). (B) Scale for FFR severity evaluation at the crown level: 0 = symptomless; 1 = slightly necrotic; 2 = moderately necrotic; 3 = severely necrotic; 4 = completely necrotic. The scale bar indicates 10 mm length.

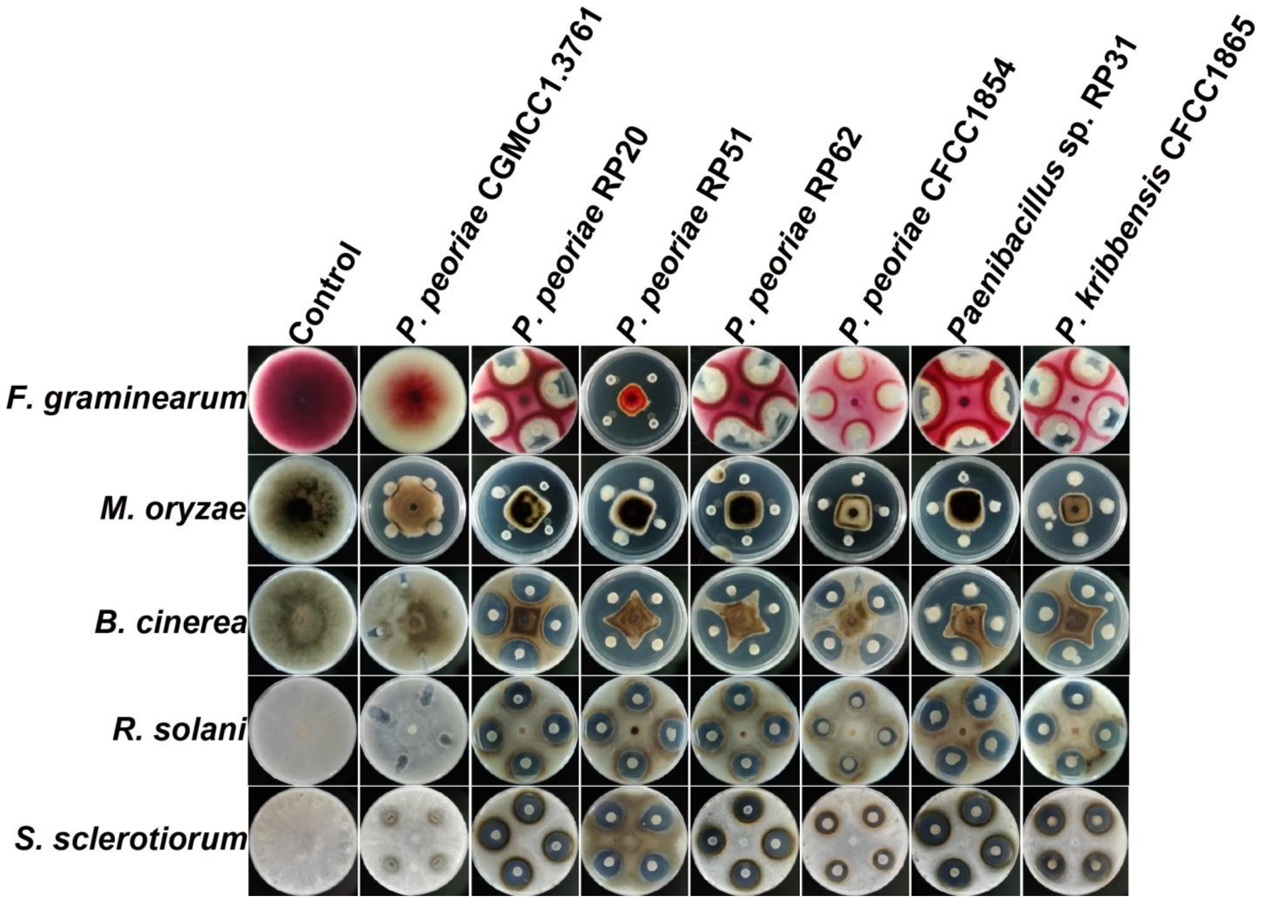

Supplementary Figure 2 Bacteria-fungi confrontation assay shows that nodule-inhabiting strains within *Paenibacillus polymyxa* complex inhibit the growth of five fungal pathogens at 7 d after inoculation on potato dextrose agar (PDA). Suspensions (5 μl) of *Paenibacillus* strains (1×10^8 CFU ml^{-1}) were inoculated at 25 mm away from the fungal plug at the center of PDA in a 90-mm Petri dish.

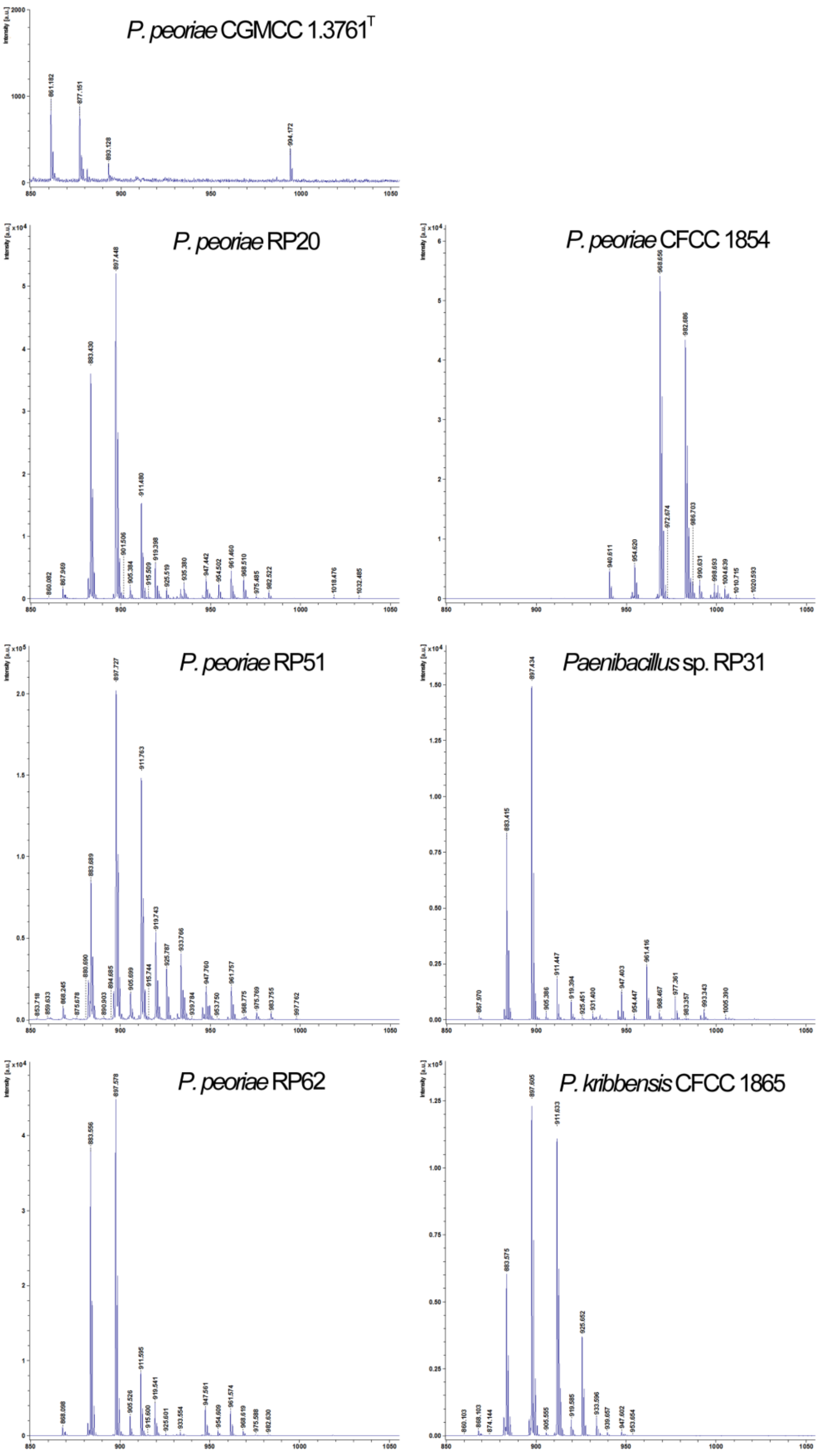

Supplementary Figure 3 MALDI-TOF mass spectra in the range of *m/z* 850–1050 show fusaricidin lipopeptides detected from whole cells of *P. peoriae* CGMCC 1.3761^{T} and nodule-inhabiting bacterial strains within the *Paenibacillus polymyxa* complex

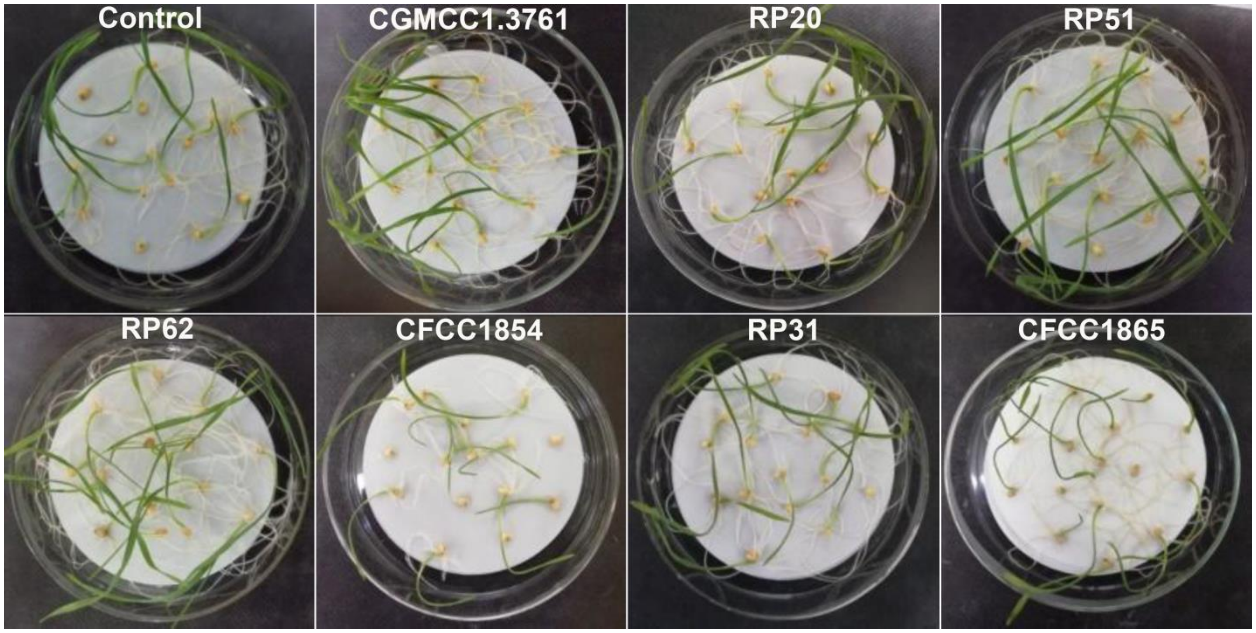

Supplementary Figure 4 Effects of *P. peoriae* CGMCC 1.3761^T and nodule-inhabiting bacterial strains within the *Paenibacillus polymyxa* complex on wheat seedling growth. Pre-germinating seeds (15 seeds per plate) were inoculated with bacterial suspension (1×10^8 CFU ml^{-1}). Control was treated with sterile water. Growth parameters were measured at 9 d after inoculation.
